# The IDentif.AI-x pandemic readiness platform: Rapid prioritization of optimized COVID-19 combination therapy regimens

**DOI:** 10.1038/s41746-022-00627-4

**Published:** 2022-06-30

**Authors:** Agata Blasiak, Anh T. L. Truong, Alexandria Remus, Lissa Hooi, Shirley Gek Kheng Seah, Peter Wang, De Hoe Chye, Angeline Pei Chiew Lim, Kim Tien Ng, Swee Teng Teo, Yee-Joo Tan, David Michael Allen, Louis Yi Ann Chai, Wee Joo Chng, Raymond T. P. Lin, David C. B. Lye, John Eu-Li Wong, Gek-Yen Gladys Tan, Conrad En Zuo Chan, Edward Kai-Hua Chow, Dean Ho

**Affiliations:** 1 The Institute for Digital Medicine (WisDM), Yong Loo Lin School of Medicine, National University of Singapore, Singapore, 117456 Singapore; 2 The N.1 Institute for Health (N.1), National University of Singapore, Singapore, 117456 Singapore; 3 Department of Biomedical Engineering, College of Design and Engineering, National University of Singapore, Singapore, 117583 Singapore; 4 Department of Pharmacology, Yong Loo Lin School of Medicine, National University of Singapore, Singapore, 117600 Singapore; 5 Cancer Science Institute of Singapore, National University of Singapore, Singapore, 117599 Singapore; 6 Defence Medical and Environmental Research Institute, DSO National Laboratories, Singapore, 117510 Singapore; 7 Infectious Diseases Translational Research Program, Department of Microbiology and Immunology, Yong Loo Lin School of Medicine, National University of Singapore, Singapore, 117545 Singapore; 8 Institute of Molecular and Cell Biology (IMCB), A*STAR, Singapore, 138673 Singapore; 9 Department of Medicine, Yong Loo Lin School of Medicine, National University of Singapore, Singapore, 119228 Singapore; 10 Division of Infectious Diseases, National University Hospital, Singapore, 119074 Singapore; 11 Department of Haematology-Oncology, National University Cancer Institute, Singapore, National University Hospital, Singapore, 119074 Singapore; 12 NUS Centre for Cancer Research (N2CR), Yong Loo Lin School of Medicine, National University of Singapore, Singapore, 117599 Singapore; 13 National Centre for Infectious Diseases (NCID), Jalan Tan Tock Seng, Singapore, 308442 Singapore; 14 Department of Laboratory Medicine, National University Hospital, Singapore, 119074 Singapore; 15 Lee Kong Chian School of Medicine, Nanyang Technological University, Singapore, 308232 Singapore; 16 Department of Infectious Diseases, Tan Tock Seng Hospital, Singapore, 308433 Singapore

**Keywords:** Viral infection, Computational biology and bioinformatics

## Abstract

IDentif.AI-x, a clinically actionable artificial intelligence platform, was used to rapidly pinpoint and prioritize optimal combination therapies against COVID-19 by pairing a prospective, experimental validation of multi-drug efficacy on a SARS-CoV-2 live virus and Vero E6 assay with a quadratic optimization workflow. A starting pool of 12 candidate drugs developed in collaboration with a community of infectious disease clinicians was first narrowed down to a six-drug pool and then interrogated in 50 combination regimens at three dosing levels per drug, representing 729 possible combinations. IDentif.AI-x revealed EIDD-1931 to be a strong candidate upon which multiple drug combinations can be derived, and pinpointed a number of clinically actionable drug interactions, which were further reconfirmed in SARS-CoV-2 variants B.1.351 (Beta) and B.1.617.2 (Delta). IDentif.AI-x prioritized promising drug combinations for clinical translation and can be immediately adjusted and re-executed with a new pool of promising therapies in an actionable path towards rapidly optimizing combination therapy following pandemic emergence.

## Introduction

COVID-19 drug development has largely focused on repurposing, either through single agent or combination therapy^1–5^. To date, clinical trial outcomes of the repurposed candidates have varied^6–8^. While many monotherapies did not mediate substantial clinical benefit, their use in effectively designed drug combinations may lead to unforeseen efficacy. Addressing this point is challenging for traditional antiviral susceptibility assays. Therefore, developing new methods that leverage unpredictable drug interactions to resolve the complexity of drug selection and dose-dependent drug synergy is essential. In fact, drug and dose selection are so tightly connected that among a pool of candidate therapies, true optimization often yields combinations of unforeseen but clinically actionable drugs and doses.

Unfortunately, simultaneous drug and dose optimization represent an insurmountable challenge. For example, 12 drugs assessed at three dosage levels results in over 500,000 possible combinations. Important strategies for synergy prediction and higher-order drug interaction analysis have been explored^9–13^. To address the challenge of ensuring clinical actionability of the combination design outcome, we developed the IDentif.AI platform, an Artificial Intelligence (AI)-based workflow for rapid combination therapy development. The first permutation of IDentif.AI used neural networks to reveal that the biological response to therapy can be represented by a smooth surface. Subsequent studies resolved this surface, which can rapidly identify optimal combinations, using a second-order algebraic function, with its coefficients determined through a small number of prospective experiments^14–26^. This correlation has subsequently been verified in prospective, human studies in infectious disease, cancer therapy, transplant medicine, and other indications^27–33^. IDentif.AI does not use pre-existing data for algorithm training, in silico modeling, or synergy prediction. Instead, it uses experimental assays to determine the drugs and doses that constitute globally optimized combination regimens. Our most recent IDentif.AI study pinpointed top-ranked combinations (based on inhibition of SARS-CoV-2-led cytopathic effects) mediated by unforeseen drug interactions^19^.

**Fig. 1:**
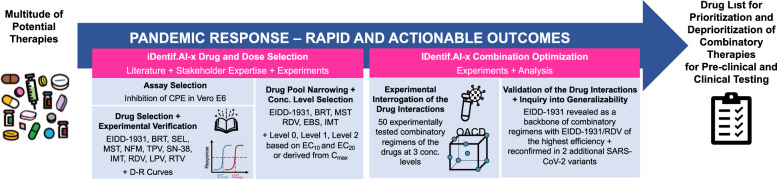
IDentif.AI-x workflow and its alignment with the pandemic response therapies prioritization. IDentif.AI-x systematically ranks drug combinations for further preclinical and potentially clinical deployment from a multitude of potential therapies. Clinical applicability considerations are integrated into IDentif.AI-x workflow to pre-emptively best position the optimized combinations for a clinical translation. EIDD-1931 (metabolite of EIDD-2801 (molnupiravir)), baricitinib (BRT), ebselen (EBS), selinexor (SEL), masitinib (MST), nafamostat mesylate (NFM), telaprevir (VX-950; TPV), SN-38 (metabolite of irinotecan), imatinib mesylate (IMT), remdesivir (RDV), lopinavir (LPV), and ritonavir (RTV) were included in the original pool in this study. CPE cytopathic effects. D-R dose-response. OACD orthogonal array composite design.

While the use of AI has facilitated a rapid identification of potential therapeutic drugs for COVID-19, in view of the rapidly evolving pandemic, and the surge in knowledge generation about drugs, targets and pathways as time passes, it is imperative that an effective AI platform can readily support a rapid response along the pandemic timeline. The desirable features of a pandemic readiness platform include technical flexibility of the AI platform and clinical acceptability of the AI-based findings. The technical flexibility of an AI platform allows it to be applied in a timely, resource-efficient, and adaptable manner at different stages of a pandemic. Moreover, the incorporation of features that improve the clinical acceptability of the AI platform, such as: involving clinical expertise in the workflow, incorporating physiological applicability, and considering practical aspects of implementation of the proposed therapies, can support rapid and broad clinical deployment of the study findings. Therefore, following the earlier demonstration^19^, we expanded the workflow and further developed the platform to its current version, IDentif.AI-x (Fig. 1). This study aimed to demonstrate IDentif.AI-x as a pandemic readiness platform by harnessing it to evaluate a starting pool of candidate therapies that have been or are being evaluated in various COVID-19 clinical settings, and in consultation with the clinical community. Candidate therapies were: EIDD-1931 (metabolite of EIDD-2801 (molnupiravir)), baricitinib (BRT), ebselen (EBS), selinexor (SEL), masitinib (MST), nafamostat mesylate (NFM), telaprevir (VX-950; TPV), SN-38 (metabolite of irinotecan), imatinib mesylate (IMT), remdesivir (RDV), lopinavir (LPV), and ritonavir (RTV) (Table 1)^4,34–45^. IDentif.AI-x implementation on a propagated, original live SARS-CoV-2 strain was completed within three weeks.

**Table 1:** Drug anti-SARS-CoV-2 efficacy and cytotoxicity when administered in monotherapy as compared to *C*_max_ obtained from the literature and regulatory documents.

Drug	EC_50_ (μM)	CC_50_ (μM)	*C*_max_ (μM)^a^	COVID-19 clinical trial
EIDD-1931	0.929	>10	11.457	NCT04575597, NCT04405570, NCT04405739, NCT04746183,
BRT	>10	>10	0.140	NCT04421027, NCT04832880, NCT04401579, NCT04891133
EBS	8.448	>10	0.00136	NCT04484025, NCT04483973
SEL	^b^	4.123	1.218	NCT04349098
MST	4.119	6.705	0.529	NCT04622865, NCT05047783
NFM	>10	>10	0.241	NCT04352400, NCT04390594, NCT04483960, NCT04623021
TPV	^b^	59.560	5.163	–
SN-38	^b^	4.784	0.143	–
IMT	6.601	27.250	2.723	NCT04394416, NCT04346147, NCT04422678
RDV	1.267	86.910	3.699	NCT04596839, NCT04292730, NCT04292899, NCT04315948
LPV	^b^	24.210	19.561	NCT04381936, NCT04315948, NCT04276688, NCT04252885
RTV	^b^	79.140	20.390	

Absolute EC_50_ and CC_50_ were obtained from the dose-response curves for each drug individually constructed based on a CPE viral assay with Vero E6 cells. EC curves were plotted after excluding %Inhibition values corresponding to drug concentrations resulting in %Cytotoxicity above 25%.

^a^Details on *C*_max_ selection for each drug are specified in Supplementary Note 2.

^b^EC_50_ was not achieved within the acceptable cytotoxicity level (below 25%).

Baricitinib (BRT), ebselen (EBS), selinexor (SEL), masitinib (MST), nafamostat mesylate (NFM), telaprevir (VX-950) (TPV), imatinib mesylate (IMT), remdesivir (RDV), lopinavir (LPV), and ritonavir (RTV).

## Results

Assay quality details for each experimental step are included in the Supplementary Note 1.

### Monotherapies were broadly not sufficiently efficacious in the actionable dosing range

The first experimental step aimed to gauge the drugs’ antiviral activities when administered as monotherapies. The dose-response (D-R) curves (Supplementary Fig. 1) revealed that the antiviral activities of the drugs were limited when they were administered as monotherapies (Table 1). RDV and EIDD-1931 were the only drugs that achieved half maximal absolute effective concentration (EC_50_) less than their maximum plasma concentration (*C*_max_) achieved in the human body with *C*_max_/EC_50_ ratios of 2.92 and 11.46, respectively. RDV, RTV and LPV performances in monotherapies were comparable with that observed in the previous IDentif.AI study based on the same assay^19^.

### IDentif.AI-x drug combination optimization

IDentif.AI-x was developed as a clinical decision support system (CDSS) for real-world application under pandemic preparedness circumstances, where experimentation is often performed under shortened timelines, as it can be executed in concert with high biosafety level laboratories and specified viral volumes processed per session. In this study, IDentif.AI-x workflow substantially reduced the time and workload needed for combination design compared to traditional methods, but the natural biological and experimental variations required incorporating an additional study team oversight process into the workflow to narrow the initial drug pool, which was performed based on each drug’s clinical acceptability, accessibility in the local context, as well as toxicity and efficacy demonstrated as a monotherapy. This additional step selected RDV, EBS, MST, IMT, BRT and EIDD-1931 to be included in a focused, six-drug experimental set that enabled the team to complete the downstream optimization process alongside laboratory guidelines while also minimizing biological and experimental variation.

The drug combinations design based on the Orthogonal Array Composite Design (OACD) table (Supplementary Table 1) considered each drug at three concentration levels (Table 2). 10% of the maximum drug concentration achieved in human blood (10% *C*_max_) identified from the published clinical studies for each drug (Table 1; Supplementary Note 2) was broadly considered as an achievable dose at the target tissue and served as the cutoff concentration level for use in the experimentations. As EC_50_ of EIDD-1931 (0.929 μM) was below its 10% *C*_max_ (1.146 μM), the maximum concentration of EIDD-1931 was further restricted to its EC_20_ to avoid overrepresentation of this drug in the experimental set and a potential saturation of the %Inhibition results (Table 2).

**Table 2:** Clinically actionable drug concentrations for the IDentif.AI-x drug combination optimization.

Drug	Level 0 (μM)	Level 1 (μM)	Level 2 (μM)
RDV	0	0.185	0.370
EBS	0	0.000068	0.000136
MST	0	0.026	0.053
IMT	0	0.136	0.272
BRT	0	0.007	0.014
EIDD-1931	0	0.315	0.458

Concentration Level 0 indicated a lack of the drug, concentration Level 1 and Level 2 were selected based on 5% and 10% *C*_max_ for RDV, EBS, MST, IMT, and BRT. Concentration Level 1 and Level 2 were selected based on absolute EC_10_ and absolute EC_20_ for EIDD-1931.

Remdesivir (RDV), ebselen (EBS), masitinib (MST), imatinib mesylate (IMT), baricitinib (BRT).

**Fig. 2:**
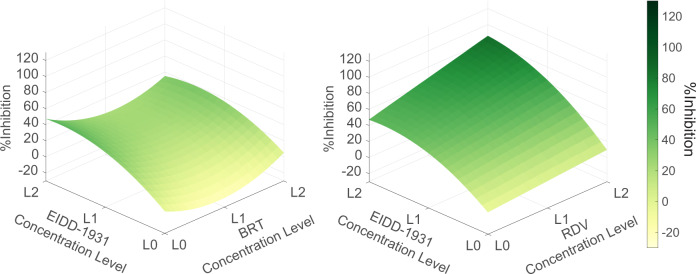
IDentif.AI-x interaction analysis. The analysis indicates that EIDD-1931 interacts differently with remdesivir (RDV) and baricitinib (BRT). EIDD-1931/remdesivir interaction surface had a convex shape indicating a synergistic interaction, while EIDD-1931/baricitinib interaction surface had a concave shape indicating a dose-dependent, mildly antagonistic interaction. L0, L1, and L2 correspond to concentration Level 0, Level 1, and Level 2.

IDentif.AI-x analysis used a quadratic equation to describe the six-drug interaction space against the SARS-CoV-2 (adjusted *R*^2^ = 0.794). The IDentif.AI-x estimated coefficients, modeling statistics and validation tests are summarized in Supplementary Table 2 and Supplementary Fig. 2. Monotherapy results demonstrated that EIDD-1931 was the most efficacious drug in the pool, even when given at EC_20_, with moderate antiviral effects. IDentif.AI-x analysis of the drug-drug interaction detected an unforeseen interaction between EIDD-1931 and RDV, which was the most efficacious combination and was predicted to achieve close to maximal %Inhibition in a synergistic or additive interaction demonstrated by the convex shape of the EIDD-1931/RDV interaction surface (Fig. 2). In addition, IDentif.AI-x-derived coefficients pointed to an interaction between EIDD-1931 and BRT (Supplementary Table 2). The EIDD-1931/BRT interaction surface had a slight concave shape across the tested BRT concentration range. BRT was predicted to have a mild antagonistic effect on the EIDD-1931-driven %Inhibition at its mid-concentration (Fig. 2). Little to no cytotoxic effects were detected in the IDentif.AI-x analysis step (Supplementary Note 3; Supplementary Data 1).

### Experimental validation of the IDentif.AI-x analysis

**Fig. 3:**
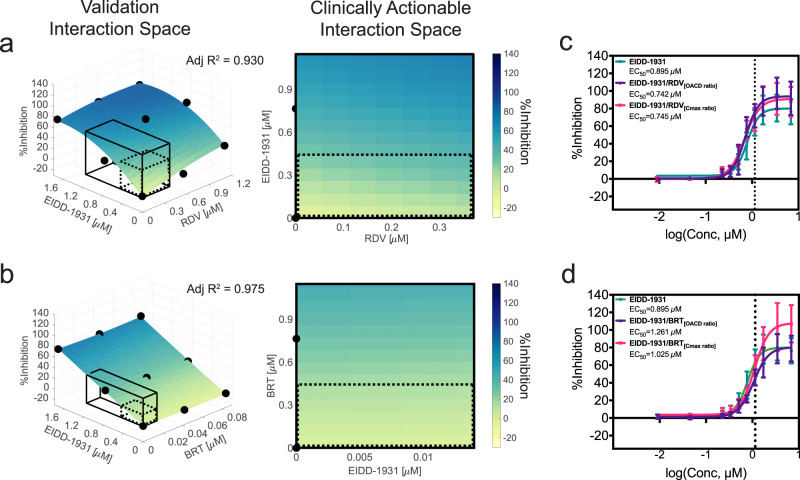
Validation of EIDD-1931 drug interactions affecting %Inhibition in the propagated, original SARS-CoV-2 strain. **a**, **b** Surface plots of EIDD-1931 interactions with remdesivir (RDV) and baricitinib (BRT) in the validation interaction space, clinically actionable interaction space (black, solid line border) and the interaction space from the IDentif.AI-x analysis (black, dotted line border). The latter two are also shown as two-dimensional maps. All experiments were performed with *N* = 3 to 4 replicates, which were independently included in the surface construction. Black, round markers indicate an average %Inhibition of the replicates for each treatment. Adjusted *R*^2^ (Adj *R*^2^) indicates goodness of the fit for each interaction surface. **c**, **d** Dose-response curves (D-R curves) of EIDD-1931 in monotherapy and in a combination with RDV and BRT at two concentration ratios: the ratio tested in the IDentif.AI-x experimental set (OACD ratio) and the ratio dictated by the *C*_max_ values of the drugs (*C*_max_ ratio). Half maximal absolute effective concentration (EC_50_) was derived from the D-R curves, which is the concentration that resulted in 50% Inhibition. The vertical line marks the 10% *C*_max_ of EIDD-1931. Please note that the EIDD-1931-only EC_50_ values (Green) were provided in both subfigures c and d to enable direct comparisons with both combinations (EIDD-1931/RDV and EIDD-1931/BRT). The entire assay was completed in one experiment, realizing all data points in a single global study, and enabling comprehensive derivation of combinations and direct comparisons between monotherapies and combinations. Error bars represent propagated standard deviation (s.d.; *N* = 3 to 4 replicates). Of note, this propagated s.d. did not arise from the replicates’ spread, but from plate-to-plate variation (s.d. of the controls). No statistically significant difference between the D-R curves was detected with sum of square F test.

In the IDentif.AI-x validation step we investigated the %Inhibition effects at different drug ratios and constructed interaction surfaces for EIDD-1931/RDV and EIDD-1931/BRT assuming a quadratic equation. With the focus on a two-drug interaction only, we recalibrated the size of the validated interaction space to range from 0 to 15% *C*_max_ of EIDD-1931 to capture the clinically actionable range (<10% *C*_max_) and the adjacent space (Fig. 3). Both EIDD-1931/RDV and EIDD-1931/BRT demonstrated ratio dependent relationships (Supplementary Fig. 3). The EIDD-1931/RDV interaction surface had a convex shape pointing to the highest %Inhibition achieved when both drugs are at their highest concentrations, suggesting it is beneficial to provide these drugs in a combination. The flat shape of the EIDD-1931/BRT interaction surface indicated that the %Inhibition results driven by EIDD-1931 were not affected by the presence of BRT. Due to an interesting multi-drug behavior observed from IDentif.AI-x analysis, we further assessed the EIDD-1931/MST combination. Although IDentif.AI-x analysis did not identify a significant interaction between EIDD-1931 and MST, it indicated that EIDD-1931/MST at maximum doses is the second most effective two-drug combination after EIDD-1931/RDV (Supplementary Data 2). After expanding the concentration range in the validation set to 15% *C*_max_ of both EIDD-1931 and MST, the concave shape of the EIDD-1931/MST interaction surface indicated that an increase in the concentrations of both drugs could mediate maximum %Inhibition (Supplementary Fig. 4A). This phenomenon, however, had the strongest effect outside of the clinically actionable range, potentially explaining why the EIDD-1931/MST interaction was not detected in the IDentif.AI-x analysis step.

Given the previously demonstrated immunomodulatory activity of BRT and synergistic potential of MST, the EIDD-1931/BRT and EIDD-1931/MST combinations can potentially be evaluated further, where dose optimization and pharmacokinetics studies may be essential to achieving optimal efficacy for clinical application.

### Dose-response curves revealed additional information for the EIDD-1931 interactions with RDV and BRT

Interaction surfaces were constructed with a small number of drug combination data points and therefore had a limited resolution. To validate IDentif.AI-x-determined EIDD-1931 interactions with RDV and BRT at a higher fidelity, in the same dataset, we included drug treatments to generate D-R curves at the two different drug ratios: as used in the OACD table and as dictated by *C*_max_.

The D-R curves revealed additional information. There was no statistical difference between D-R curves (*p* = 0.0513); however, we observed a slight shift in the D-R curves for both combinations: towards a lower and higher EIDD-1931’s absolute EC_50_ for EIDD-1931/RDV and EIDD-1931/BRT, respectively (Fig. 3c, d; Supplementary Fig. 5). The mild antagonistic effect of BRT at the OACD ratio was consistent with the IDentif.AI-x analysis. Overall, these results suggest small effect sizes of the tested interactions of EIDD-1931. Interestingly, at high concentrations, the D-R curve shapes revealed a potential boost in maximum %Inhibition achievable by EIDD-1931 co-administered with RDV at both ratios, and by EIDD-1931/BRT_[Cmax ratio]_ (Fig. 3c, d; Supplementary Fig. 5). This phenomenon was not observed for EIDD-1931/BRT_[OACD ratio]_ which, instead, was shown to induce a mildly antagonistic shift in EC_50_.

The potentially synergistic or additive efficacy interaction demonstrates that combining EIDD-1931 with either RDV or BRT at the right ratio achieved a higher efficacy than each drug alone, which highlights the potential of EIDD-1931 serving as a backbone to combinational therapies against the SARS-CoV-2. However, as the potential beneficial interactions were detected outside of the actionable interaction space, additional dosing strategies may need to be considered to optimize these interactions in a clinical setting.

### The efficacy of the pinpointed therapies against SARS-CoV-2 B.1.351 and B.1.617.2 variants

**Fig. 4:**
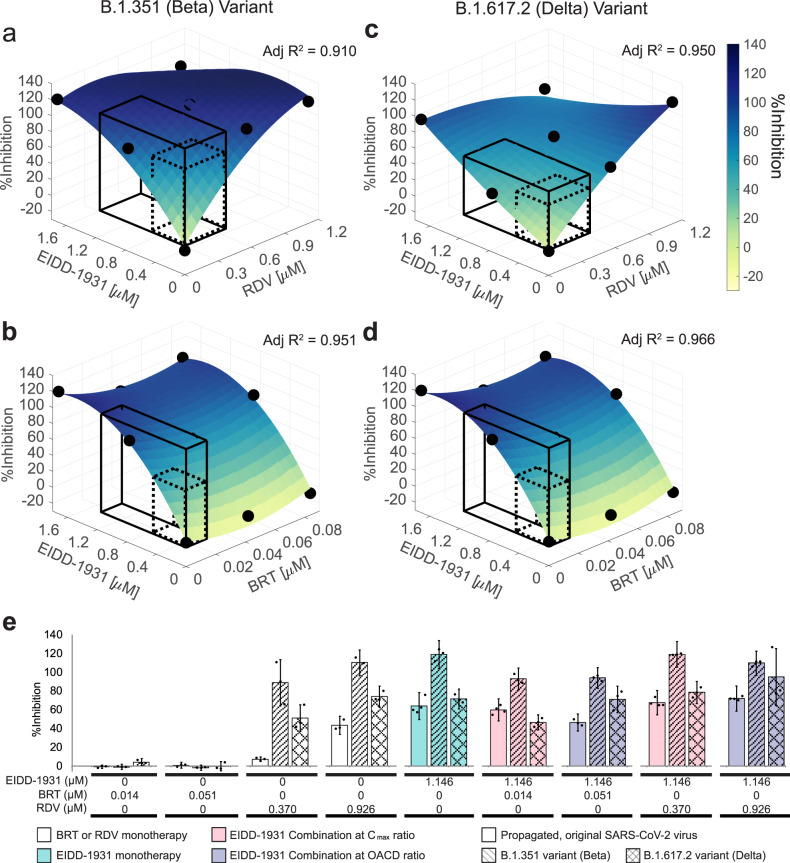
Validation of EIDD-1931 drug interactions affecting %Inhibition in SARS-CoV-2 B.1.351 and B.1.617.2 variants. **a**–**d** Surface plots of EIDD-1931 interactions with remdesivir (RDV) and baricitinib (BRT) in the validation interaction space, clinically actionable interaction space (black, solid line border) and the interaction space from the IDentif.AI-x analysis (black, dotted line border). All experiments were performed with *N* = 3 replicates, which were independently included in the surface construction. Black, round markers indicate an average %Inhibition of the replicates for each treatment. Adjusted *R*^2^ (Adj *R*^2^) indicates goodness of the fit for each interaction surface. The experiments with SARS-CoV-2 B.1.351 variant (**a**, **b**), and B.1.617.2 variant (**c**, **d**) were performed in two independent sets. **e** %Inhibition against the propagated, original SARS-CoV-2 strain (bars with block filling), B.1.351 variant (bars with line filling) and B.1.617.2 variant (bars with cross lines filling) of 10% *C*_max_ EIDD-1931 in monotherapy (green) and in a combination with RDV and BRT at two concentration ratios: the ratio dictated by the *C*_max_ values of the drugs (C_max_ ratio; pink) and the ratio tested in the IDentif.AI-x experimental set (OACD ratio; purple). Black markers indicate individual data points. Error bars represent propagated standard deviation (s.d). Of note, this propagated s.d. did not arise from the replicates’ spread, but from plate-to-plate variation (s.d. of the controls). No statistically significant difference was detected with Kruskal–Wallis test when followed by Dunn’s post hoc test.

We retested the efficacy of the pinpointed monotherapies and combination treatments against the SARS-CoV-2 B.1.351 and B.1.617.2 variants (Fig. 4 and Supplementary Figs. 4B, C and 6). When tested against B.1.351 variant, EIDD-1931 and RDV monotherapies demonstrated an increased antiviral activity as compared to the propagated, original strain (Supplementary Fig. 6). Accordingly, the EIDD-1931 interaction surfaces demonstrated saturation regions at high concentrations of EIDD-1931 and RDV (Fig. 4a). When tested against B.1.617.2 variant, EIDD-1931 retained its high antiviral activity, while RDV demonstrated an increased antiviral activity as compared to the propagated, original strain (Fig. 4; Supplementary Fig. 6). Similar to the propagated, original strain, the effects of EIDD-1931 combinations depended on the ratio in which the drugs were combined. Overall, the experiments with SARS-CoV-2 B.1.351 and B.1.617.2 variants confirmed EIDD-1931 for consideration as a monotherapy and as a backbone of combinatory treatment against SARS-CoV-2. Dose adjustments in combination therapy should be performed for each specific variant.

### Cytotoxicity of EIDD-1931 in the interactions

**Fig. 5:**
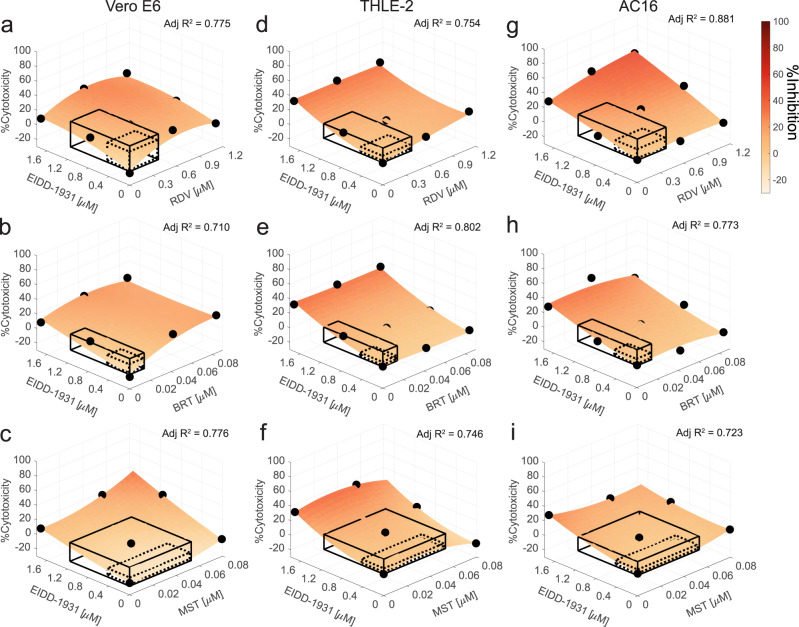
Validation of EIDD-1931 drug interactions affecting %Cytotoxicity. Surface plots of EIDD-1931 interactions with remdesivir (RDV), baricitinib (BRT) and masitinib (MST), in the validation interaction space, clinically actionable interaction space (black, solid line border) and the interaction space from the IDentif.AI-x analysis (black, dotted line border), based on the experimentation in Vero E6 cells (**a**–**c**), THLE-2 (**d**–**f**) and AC16 (**g**–**i**). **a**–**i** All experiments were performed with *N* = 3 to 4 replicates, which were independently included in the surface construction. Black, round markers indicate an average %Cytotoxicity of the replicates for each data point. Adjusted *R*^2^ (Adj *R*^2^) indicates goodness of the fit for each interaction surface.

When %Cytotoxicity was tested in Vero E6, only a narrow range of readings (−8.7 ± 4.9% to 9.6 ± 9.3%) was detected, which may have contributed to a low goodness-of-fit of the quadratic model (adjusted *R*^2^ = 0.017) and the IDentif.AI analysis not detecting any significant interactions between the drugs (Supplementary Fig. 7 and Supplementary Table 3) in the clinically actionable range. Nevertheless, in the validation stage, we interrogated EIDD-1931/RDV, EIDD-1931/BRT, EIDD-1931/MST interactions’ effects on %Cytotoxicity (Supplementary Fig. 8). EIDD-1931/RDV and EIDD-1931/BRT had a convex shape (Fig. 5a, b) while the EIDD-1931/MST had a concave shape (Fig. 5c) indicating that %Cytotoxicity is a result of an interaction between EIDD-1931 and the drugs. However, %Cytotoxicity was not predicted to expand beyond 23% for any of the drug combinations in the actionable range.

To gauge the potential cytotoxicity that may be observed in clinical settings, we investigated cytotoxic effects of the EIDD-1931 drug combinations in cell lines of human origin: liver epithelial cells (THLE-2) and cardiomyocytes (AC16) (Fig. 5d–i; Supplementary Fig. 8). The interaction surfaces had different shapes in different cell lines, highlighting the target-specific cytotoxic characteristics of the treatments. The regular shape of each interaction surface with uniformly high %Cytotoxicity tested at high EIDD-1931 concentration independent of the presence of the other drugs indicates that cytotoxicity in THLE-2 was driven by EIDD-1931 and it was not significantly affected by its interactions with RDV, BRT and MST (Fig. 5d–f). In AC16 cells, MST did not increase EIDD-1931-driven cytotoxicity; BRT mildly alleviated it in a ratio-dependent fashion; and RDV demonstrated dose-dependent cytotoxic increase, with predicted 29% maximum %Cytotoxicity in the clinically actionable interaction space (Fig. 5g–i).

## Discussion

Our study pinpoints EIDD-1931 to be a promising therapeutic for COVID-19, both as a monotherapy and as a backbone drug for combination therapies. In addition to the specific combinations containing EIDD-1931 that emerged from the study, this work also points to the potential of classes of therapies that may be suitable for administration in combination with EIDD-1931, such as immunomodulatory agents or protease inhibitors, among others. In line with previous reports of EIDD-2801 (molnupiravir; EIDD-1931’s prodrug)^46,47^, our low toxicity results also suggest that EIDD-1931 may be well tolerated. However, additional studies addressing longer-term toxicity or suitability for specific patient populations may need to be conducted. Furthermore, dose-dependent interaction findings from our study indicate a potential need to further explore suitable clinical dosing strategies for combination regimens. Taken together with the fact that EIDD-1931 can be administered orally^47^, our findings support the potential of the drug as a rapidly deployable therapy. EIDD-1931 is hypothesized to inhibit viral replication by inducing lethal mutagenesis in coronaviruses^4^. EIDD-2801 was initially shown to inhibit SARS-CoV-2 in primary human airway epithelial cell cultures and in multiple animal models^4,40,48,49^, and recently in mild-to-moderate COVID-19 patients^50^. The interim analysis of a Phase 2/3 trial MK-4482-002 (NCT04575597) reported that EIDD-2801 reduced the risk of hospitalization or death in COVID-19 adult patients by approximately 50%^51^, which prompted the drug to be approved by the United Kingdom’s Medicines and Healthcare products Regulatory Agency (MHRA)^52^. Upon completion, the trial MK-4482-002 demonstrated that the EIDD-2801’s relative risk reduction effect was 30%^53^. On December 23rd, 2021, the United States Food and Drug Administration (US FDA) granted emergency use authorization (EUA) of EIDD-2801 for individuals with mild to moderate COVID-19 who are at high risk of becoming severely ill^54^. However, current findings suggest that it may be important to further explore the identification of optimal parameters within which molnupiravir can be delivered in combination with other classes of therapies and that they may also be suitable for further preclinical and/or clinical evaluation with careful dose adjustment and optimization strategies to maximize synergistic efficacy at minimal toxicity. These findings may also be applicable towards downstream combination regimens containing other repurposed and/or novel therapies, as well as clinical trial designs to evaluate these regimens.

Furthermore, it should be noted that IDentif.AI-x pinpointed top-ranked EIDD-1931 independently from the aforementioned clinical trial data as IDentif.AI-x does not require clinical data to implement its optimization processes, which are conducted with the live virus. This is also true for lower-ranked drug combinations and monotherapies. In fact, similar to previous IDentif.AI findings^19^, IDentif.AI-x independently replicated failed monotherapies and established drug-drug interactions reported in clinical trials. For example, IDentif.AI-x identified limited efficacy of RDV and IMT as monotherapy candidates against SARS-CoV-2, similar to previously reported clinical findings^55–57^. Interestingly, IDentif.AI-x revealed that adding BRT to RDV moderately improved the therapy’s rank from 521/729 to 454/729 (Supplementary Data 2). While our study focused on antiviral drug properties, and did not capture immunomodulatory effects of BRT, this is in line with findings from the Adaptive COVID-19 Treatment Trial 2 (ACTT-2) report, in which BRT in combination with RDV was superior to RDV alone in reducing recovery time and accelerating improvement in clinical status in COVID-19 patients^8^. Furthermore, IDentif.AI-x also revealed the unforeseen interaction between EIDD-1931 and RDV without any clinical data using a lean, streamlined AI methodology. Schultz and colleagues have recently and independently identified an additive interaction between the EIDD-1931 and RDV using traditional, large-scale screening methodologies^58^. Collectively, these results demonstrate the potential actionability of IDentif.AI-x as an effective go/no-go platform for prioritizing and advancing combination therapies towards further preclinical or widespread clinical deployment.

Overall, this work demonstrates IDentif.AI-x’s potential as a pandemic readiness platform to rapidly prioritize drug combinations for further consideration based on high efficacy, and de-prioritize combinations that may be avoided due to lack of efficacy as optimized to a specific SARS-CoV-2 variant. A key technical attribute of IDentif.AI-x is that its combination design workflow efficiently investigated the interaction space of six drugs at three concentration levels (mounting to 729 drug-dose combination) using only 50-drug combinations. The whole workflow was completed within three weeks—a staggering speed—while also optimizing resource utilization, which are important considerations given the time criticality of a pandemic and the inevitable resource constraints following the onset of a pandemic. Another advantage of IDentif.AI-x is that it relies on experimentally procured data of phenotypic response—%Inhibition or %Cytotoxicity. The data collection process does not require extensive domain knowledge of drugs, host, and disease mechanisms while it allows to ensure control over the quality and completeness of the data inputs into the algorithm. This technical feature allows IDentif.AI-x to often yield efficacious drug combinations with unforeseen drug-drug interactions for further testing with a high technical reliability. Furthermore, the simplicity and low resource requirements facilitate IDentif.AI-x workflow to be readjusted and reapplied with a new drug pool as the high-potential drugs emerge over time. A platform that can rapidly identify treatments for prioritized testing will also be beneficial in the emergence of new viral variants such as B.1.351 (Beta) and B.1.617.2 (Delta) variants, which potentially affect vaccination and therapeutic efficacies, and also for the treatment of patients that could not be vaccinated, including those who remain ill with evidence of sustained viral replication.

A crucial component of the IDentif.AI-x as a pandemic readiness platform was the involvement of stakeholders’ expertise in the workflow: from initial clinician consultation for the drug pool selection, to understanding how clinicians prioritize combination regimens for rapid clinical deployment. The restriction of the drugs in the pool from 12 to six drugs was also driven by an alignment with the regulations minimizing the operator’s exposure to the virus and minimizing experimental variation. The 12-drug pool was narrowed down according to the drugs’ clinical acceptability, accessibility in the local context, as well as toxicities and efficacies demonstrated as monotherapies. In addition, the drug doses for testing were selected with consideration of each drug’s *C*_max_/EC_x_ ratio to avoid pinpointing drug combinations outside clinically actionable concentrations, thereby improving the clinical acceptability of IDentif.AI-x findings. Another key technical consideration added to the IDentif.AI-x workflow to improve its clinical acceptability was the use of higher-resolution OACD to generate a ranked list, with high estimation efficiency of the second-order drug-dose interaction space and drug-drug interactions, including those of lower-ranked combinations. Drug combinations of comparable efficacy with fewer drugs or lower drug concentrations are more clinically actionable, in terms of streamlined market access, regulatory clearance and potential to prevent drug resistance from developing. Additionally, IDentif.AI-x can be tailored to generate combinations that address supply chain considerations and local regulations to make the most out of what is available in the geographical and economic feasibility context for a pandemic readiness program that is inclusive of low-and middle-income countries (LMICs).

It is important to note that this study was conducted in an in vitro live virus model. While the in vitro model may be a limitation in interpreting the result for clinical decision-making, in vitro findings are crucial first steps in exploring the potential efficacy, safety, and unforeseen drug interactions of any proposed repurposed drug combinations, especially with a novel virus. It is critical for the pinpointed combinations from this in vitro study to be further evaluated in subsequent in vivo and clinical dose optimization studies prior to broad clinical deployment. This study evaluated a pre-specified drug pool, and further studies with additional drug candidates are warranted. Additionally, although derived from the input of local infectious disease clinicians, in this study we interrogated a small pool of handpicked drugs. Developing a set of drug selection criteria such as drug class, administration route, prior evidence of interaction with other drugs, clinical relevance and accessibility may streamline the selection of the drug pool. Furthermore, while the current study only examined the effects detectable in our specific experimental model and, for example, did not incorporate the immunomodulatory effects of the anti-inflammatories (SN-38, BRT), future work using applicable assays towards combination therapy development with immunomodulators and drugs targeting other specific infection pathways/mechanisms is warranted as IDentif.AI-x can be implemented in virtually all assays, provided quantifiable efficacy and toxicity readouts are available. Including immunomodulation will potentially create viable therapeutic options for severe patients as shown by recent clinical progress^7^.

The IDentif.AI-x workflow also has some technical limitations. It is developed for rapid optimization and clinical actionability, and complementary strategies can be integrated to address them. First, the IDentif.AI-x interaction space interrogation assumes a quadratic relationship with the efficacy/cytotoxicity responses. The optimized combinations presented here are largely limited to two-drug combinations, rapidly identifying the most significant drugs and their partners from a large drug combination search set. Further development of more complex combinatorial therapy strategies, such as four- or five-drug combinations would likely require some reconciliation of higher-order interactions, similar to previous studies^9^. Second, only limited dosage ratios were tested. The observation that the same drug combination at two different ratios can potentially exhibit the opposite interactions points out the importance of optimizing drug doses at the same time as their combinations. Nevertheless, the current results suggest that further preclinical and clinical dosing optimization may reveal the full potential of the pinpointed combination therapies in terms of their synergistic potency (i.e., beneficial dose reduction) and synergistic efficacy interactions (i.e., beneficial increase in maximum efficacy). Additional correlation studies with clinical trial outcomes, when available, will also further determine the applicability of IDentif.AI-x towards go/no-go decisions on combination regimens pinpointed by IDentif.AI-x. The IDentif.AI-x process in its current form has resulted in promising outcomes, further development of IDentif.AI-x and its potential integration with other methodologies may further enhance its clinical relevance.

Clinical decision making in response to the COVID-19 pandemic has been dynamically adapting to new information^59,60^. With new evidence emerging, Infectious Diseases Society of America (IDSA) COVID-19 treatment guidelines provide updated recommendations for certain monotherapies and combinations depending on severity and setting^61^. Dose optimization has been increasingly recognized as a key therapy optimization element for maximizing public health benefits from the therapeutic solutions of limited supply^62^. Interestingly, a wide range of AI-based applications have been deployed since the onset of the pandemic, that have significantly accelerated drug development and identified several monotherapy candidates that have entered clinical trials or even have been approved for use against COVID-19. These include the BenevolentAI knowledge graph that identified BRT (FDA EUA for use alone or with remdesivir)^63^; AI-based network analysis by Gysi and co-authors that suggested nelfinavir (ongoing clinical trial, JPRN-jRCT2071200023) and dexamethasone (EMA approval)^64^; and AI-based Molecular Transformer-Drug Target Interaction (MT-DTI) model that suggested RDV (FDA EUA)^65^; among others. In addition to single-agent therapy development, it is also important to pinpoint combination regimens that are clinically actionable, both in composition and dosing parameters based on available recommendations. Drug combinations could be more efficacious, safer, less toxic and may even be readily available than new single-agent therapies. The IDentif.AI-x platform reported here could provide key insights and address gaps regarding how to optimally combine the therapies.

This work reports the application of IDentif.AI-x towards the rapid optimization and prioritization of combination therapy regimens against COVID-19. The IDentif.AI-x optimization process pinpointed EIDD-1931/RDV, EIDD-1931/BRT and EIDD-1931/MST as regimens that may be suitable for further evaluation and development. IDentif.AI-x did not rely on detailed scientific literature networks, pre-existing databases, or in silico modeling to design these combinations. Instead, it harnessed data from carefully designed drug-dose permutations and prospectively executed studies to drive the optimization process to complement existing strategies in the fight against the COVID-19 pandemic. The promising findings from this work support the expansion of IDentif.AI-x towards a broad range of applications in addressing antimicrobial resistance as well as optimizing intervention using antiviral, antibiotic, and antifungal therapies.

## Methods

### Starting drug pool

We selected 12 drugs based on their antiviral potential, actionability and technical factors. Efficacy potential was based on evidence emerging from existing literature and clinical trials. The actionability was judged by the drug potential—on its own and in combination—to be deployed in a clinical setting, considering its accessibility, safety profile, administration route, current clinical practice, among others. Technical factors included assessing if the experimental model was compatible with the hypothesized action mechanism of the drug reported in the literature. The 12 candidate drugs had hypothesized mechanisms of either inhibiting SARS-CoV-2 entry into the host cell—BRT, NFM, IMT—or inhibiting SARS-CoV-2 replication—EIDD-1931, EBS, SEL, MST, TPV, SN-38, RDV, LPV, and RTV^4,35–37,41–45^.

EIDD-1931 (Selleck Chemicals, Cat#S0833), nafamostat mesylate (NFM; Selleck Chemicals, Cat#S1386) and imatinib mesylate (IMT; Selleck Chemicals, Cat#S1026) were dissolved in sterile-filtered water. Baricitinib (BRT; Selleck Chemicals, Cat#S2851), ebselen (EBS; Selleck Chemicals, Cat#S6676), selinexor (SEL; Selleck Chemicals, Cat#S7252), masitinib (MST; Selleck Chemicals, Cat#S1064), telaprevir (TPV; Selleck Chemicals, Cat#S1538), SN-38 (Selleck Chemicals, Cat#S4908), remdesivir (RDV; Selleck Chemicals, Cat#S8932), lopinavir (LPV; Selleck Chemicals, Cat#S1380) and ritonavir (RTV; Selleck Chemicals, Cat#S1185) were dissolved in DMSO (MP Biomedicals, Cat#0219605590).

### SARS-CoV-2

All experiments with a live virus were conducted in a biosafety level-3 (BSL-3) laboratory. Severe acute respiratory syndrome coronavirus 2 (SARS-CoV-2) was previously isolated from a nasopharyngeal swab in early 2020 in Singapore^66^ and has undergone several rounds of propagation to form SARS-CoV-2 original, propagated variant used in this study (virus source: Biological Defense Program, DSO National Laboratories). This propagated variant was identified to have genetic mutations as compared to the original strain. The second viral strain—the B.1.351 (Beta) variant—was isolated from a nasopharyngeal swab in early 2021 in Singapore and was registered in GISAID EpiFlu™ Database under hCoV-19/Singapore/239/2021 (virus source: National Public Health Laboratory, NCID). The third viral strain—the B.1.617.2. (Delta) variant—was isolated from a nasopharyngeal swab in 2021 in Singapore (virus source: National Public Health Laboratory, NCID). Each viral strain was propagated in Vero E6 C1008 cells in maintenance medium containing minimum Eagle’s medium (MEM; Gibco, Cat#11095-080) with 2% heat-inactivated fetal bovine serum (HI-FBS; Gibco, Cat#10082147), Penicillin/Streptomycin (Gibco, Cat#15140-122), Sodium pyruvate (Gibco, Cat#11360-070), Sodium bicarbonate (Gibco, Cat#25080-094) and non-essential amino acid (Gibco Cat#11140-050). Viral ToxGlo™ Assay (Promega, Cat#G8943) was used to determine the virus titer by a standard tissue culture infectious dose (TCID_50_) endpoint dilution assay and luminescence readout with a microplate reader (BioTek).

### Cell cultures

African green monkey kidney Vero E6 C1008 cells were cultured in MEM supplemented with 10% HI-FBS prior to use for the infection assay and were subsequently added in 96-well white plates (Greiner Bio-One, Cat#655074) at a density of 2 × 10^4^ cells/well.

The cultivation of the human liver epithelial THLE-2 cells (ATCC, Cat#CRL-2706) required a coating medium consisting of bronchial epithelial basal medium (BEGM Bullet Kit, Cat#CC-3170) with human fibronectin (0.01 mg/mL; Biological Industries, Cat#03-090-1), bovine collagen Type I (0.03 mg/mL; Stem Cell Technologies, Cat#07001) and bovine serum albumin (0.01 mg/mL; Sigma-Aldrich). THLE-2 cells were plated in pre-coated 96-well plates at 3 × 10^3^ cells/well density and cultured in bronchial epithelial cell growth medium (BEGM Bullet kit; Lonza, Cat#CC-3170) excluding gentamicin/amphotecirin and epinephrine, but supplemented with 10% FBS (Biowest, Cat#S1300), human epidermal growth factor (5 ng/mL, Peprotech, Cat#AF-100-15) and phosphoethanolamine (70 ng/mL, Sigma, Cat#P0503).

AC16 human cardiomyocytes (Millipore, Cat#SCC-09) were plated in 96-well plates at 2 × 10^3^ cells/well density and cultured in DMEM/F12 (Life Technologies, Cat# 11320033) mixed with 12.5% FBS (Biowest, Cat# S1300), and 1% penicillin-streptomycin (Life Technologies, Cat# 15140122). All cell cultures were incubated at 37 °C in a humidified atmosphere containing 5% CO_2_.

### Viral inhibition and cell cytotoxicity of drugs

All experiments with the live SARS-CoV-2 (the propagated, original strain, B.1.351 and B.1.617.2 variants) were performed in a BSL-3 laboratory. Each treatment was prepared in the culture media and pipetted into the wells of the white 96-well plate in triplicate. 2 × 10^4^ Vero E6 C1008 cells were added into each well with and without SARS-CoV-2 (100 TCID_50_) to obtain %Inhibition of the virus-induced cytopathic effect (CPE) and the drug toxicity-induced CPE (%Cytotoxicity), respectively. The maximum DMSO concentration used in each experimental step and media only served as vehicle controls. Plates were incubated for 72 h before measuring the cell viability via Viral ToxGlo™ per the manufacturer’s instructions. Drug cytotoxicity and viral CPE inhibition were calculated, as described previously^19^. The %Inhibition and %Cytotoxicity were derived in independent biological replicates based on different activity ranges, so their effect sizes are not directly comparable. In case no difference was detected between the vehicle and media only controls, the results from these treatments in each plate were pooled together and served as plate-specific control used in the calculations. The calculations used in the validation experimental step used an average of pooled measurements from the control treatments from all plates. GraphPad Prism 9 software (GraphPad Software) was used to plot D-R curves and to derive absolute effective concentrations EC_10_, EC_20_ and EC_50_ of %Inhibition and absolute cytotoxic concentrations CC_50_ of %Cytotoxicity.

%Cytotoxicity in the validation experimental step was calculated in THLE-2 human liver and AC16 human cardiomyocyte cell lines. The drugs were added to the wells after the cells were allowed to adhere to the surface for 24 h. The plates were incubated for 72 h before measuring the cell viability via CellTiter-GLO (Promega, Cat#G7570) per the manufacturer’s instructions. %Cytotoxicity calculations in THLE-2 and AC16 were performed using an average of pooled measurements from the control treatments from all plates.

### Drugs as monotherapy candidates

The concentration range for each drug in the first experimental step was prepared by a serial dilution with a dilution factor of three: 1.9 × 10^−^^6^ µM to 10 µM for EIDD-1931, BRT, EBS, NFM and SN-38; 1.9 × 10^−5^ µM to 100 µM for SEL, MST, TPV, IMT, and RDV; 1.1 × 10^−^^3^ µM to 200 µM for LPV and RTV.

Vero E6 cells were exposed with and without the live virus to an increasing concentration of each drug on its own, constructed dose-response (D-R) curves and calculated EC_50_—the drug concentration at which half of the viral-induced CPE is inhibited. An analogical process was performed to understand at what concentration each drug became cytotoxic.

Importantly, to ensure the clinical acceptability of the findings, the concentration range tested for each drug was selected with consideration of their *C*_max_ achieved in the human body (Table 1) to capture the efficacy in a concentration range of interest—clinically actionable concentrations with potential human efficacy. A high *C*_max_/EC_50_ ratio indicates a drug’s capability to reach the concentrations in the human blood plasma that is sufficient to provide antiviral efficacy^67^. The specifics of the *C*_max_ selection for each drug are presented in the Supplementary Note 2.

### Drug interaction analysis in the IDentif.AI-x experimental step

In the IDentif.AI-x experimental step, a set of curated drug combinations consisting of three concentration levels (Level 0, Level 1, and Level 2) for a six-drug library was designed in accordance with the OACD as described by Xu et al.^68^. Specifically, Level 0 indicated the absence of the drug and Level 1 and Level 2 corresponded to two clinically actionable concentrations of each drug, selected based on the D-R curves and *C*_max_ values. This six-drug OACD was generated by combining a resolution VI 32-run two-level fractional factorial and an 18-run three-level orthogonal array. These 50 runs formulated the minimum amount of experimental drug combinations required to screen each drug’s effects through their linear, bilinear (drug-drug interactions), and quadratic parameters. The resolution VI six-drug OACD is tabulated in Supplementary Table 1.

IDentif.AI-x analysis correlated the six-drug in vitro experimental data into a quadratic series to elicit optimized drug combinations and drug-drug interactions. The analysis was performed in MATLAB R2020a (Mathworks, Inc.)^19^. IDentif.AI-x analysis derived two quadratic series—%Inhibition, %Cytotoxicity—by including all experimental replicates as inputs and performing bidirectional elimination in which the *P* value from the F-statistic served as the removal criterion. Box-Cox transformation determined appropriate transformations to improve the residual distributions and the goodness of the fit represented by adjusted *R*^2^. Computational validation tests including residual analysis and outlier analysis were performed for each of the two IDentif.AI-x-derived series.

### Drug interaction analyses in the validation experimental step

The combinations for further evaluation were selected not only based on their antiviral efficacy, but also on the predicted implementation capability and clinical acceptability. Drug combinations with fewer drugs are more actionable due to more streamlined market access, regulatory clearance, administration, and minimal interactions with concomitant drugs, among others. Additionally, it has been demonstrated that the patients are more likely to adhere to drug regimens that have a smaller number of pills. Given the above, 2-drug combinations were prioritized. Interaction surfaces were constructed using drug combinations selected via D-optimal experimental design (*N* = 9 treatments for EIDD-1931/RDV and EIDD-1931/BRT; *N* = 6 treatments for EIDD-1931/MST; 3–4 replicates per treatment) performed in MATLAB R2020a (MathWorks, Inc.). The concentration ranges for constructing interaction surfaces were set as: 0—1.719 μM for EIDD-1931; 0—1.10 μM for RDV; 0—0.084 μM for BRT and 0—0.079 μM for MST to include the concentration ratios explored in the IDentif.AI-x experimental step at the high EIDD-1931 concentrations. We assumed a quadratic model of the drug interactions. All replicates were included in the construction of the surfaces.

GraphPad Prism 9 software (GraphPad Software) was used to plot D-R curves and derive EC_50_ of %Inhibition and CC_50_ of %Cytotoxicity of the validation set treatments (monotherapies and combinations). Drug combinations were tested at two fixed ratios: (i) Level 2/Level 2 ratio for EIDD-1931/RDV, and Level 1/Level 2 ratio for EIDD-1931/BRT from IDentif.AI-x experimental set (OACD ratio); and (ii) *C*_max_/*C*_max_ ratio (*C*_max_ ratio). %Cytotoxicity was evaluated in terms of its effects on the %Inhibition assay.

### Statistical analyses

All in vitro experiments were performed in at least three biological replicates. %Inhibition and %Cytotoxicity are presented as mean ± propagated standard deviation (s.d.):

1 $$\sigma _I^2 = \left( {\frac{{\partial I}}{{\partial E_ - }}} \right)^2\sigma _{E_ - }^2 + \left( {\frac{{\partial I}}{{\partial c_ - }}} \right)^2\sigma _c^2 + \left( {\frac{{\partial I}}{{\partial c_ + }}} \right)^2\sigma _{c_ + }^2$$

2 $$\sigma _T^2 = \left( {\frac{{\partial T}}{{\partial c_ + }}} \right)^2\sigma _{c_ + }^2 + \left( {\frac{{\partial T}}{{\partial E_ + }}} \right)^2\sigma _{E_ + }^2$$

In Eqs. 1 and 2, σ_T_ and σ_I_ represent the propagated s.d. for the mean value of %Cytotoxicity and %Inhibition, respectively. The equations consider the spread of the raw luminescence signals of the positive control (control cells), negative control (cells + virus control), and the experimental triplicates with and without virus (cell + drugs + virus and cells + drugs), which are represented by σ_c+_, σ_c−_, σ_E+_, and σ_E−_ respectively^69^.

D-R curves were compared using a sum of squares F test. The IDentif.AI-x-estimated coefficients were analyzed using sum of squares F-test and *P*-values for each individual coefficient obtained from stepwise regression. Sample distribution was tested with Shapiro–Wilk normality test. The Kruskal–Wallis test by ranks was used for multiple comparisons, followed by Dunn’s post hoc test for pairwise comparisons. For two-group comparisons, Student’s two-tailed t-test and Wilcoxon rank-sum test were used for normally and non-normally distributed populations, respectively. Bonferroni correction was used in multiple comparisons. Alongside the *P*-values, the results were interpreted in the light of logic, background knowledge and the specifics of the experimental design^70^.

### Reporting summary

Further information on research design is available in the Nature Research Reporting Summary linked to this article.

## Supplementary information

Supplementary Information

Supplementary Data 2

Supplementary Data 3

Supplementary Data 4

Reporting Summary

## Data Availability

All data generated and analyzed during this study are included in this published article and its supplementary information. The %Inhibition and %Cytotoxicity data together with the code for the six-drug IDentif.AI-x drug combination optimization step, can be found in the Supplementary Data 1. All %Inhibition and %Cytotoxicity predictions generated in IDentif.AI analysis can be found in Supplementary Data 2. The experimental data underlying the monotherapy and validation analyses can be found in Supplementary Data 3 and 4, respectively.

## Code availability

Data processing and IDentif.AI-x analyses in this study were conducted using basic functions written in Python programming language and in Matlab R2020a (Mathworks, Inc.). IDentif.AI-x analyses were conducted using the built-in “stepwiselm” function in Matlab R2020a as provided in Supplementary Data 1. The custom codes can be shared on reasonable request to the corresponding authors.
