# Supplementary material for: The IDentif.AI-x pandemic readiness platform: Rapid prioritization of optimized COVID-19 combination therapy regimens
Source: NPJ Digit Med. 2022 Jun 30;5:83. doi: 10.1038/s41746-022-00627-4 (PMC9244889; doi:10.1038/s41746-022-00627-4)
Supplement: Supplementary file 1 — Supplementary Information [file 41746_2022_627_MOESM1_ESM.pdf]

The IDentif.AI-x Pandemic Readiness Platform: Rapid Prioritization of Optimized COVID-19 Combination Therapy Regimens

This Supporting Information includes:

1. Supplementary Note 1 to 3
2. Supplementary References
3. Supplementary Figures 1 to 8
4. Supplementary Tables 1 to 3
5. Supplementary Data 1-4

*Supplementary Data 2-4 are included as separate files

1. Supplementary Notes

Supplementary Note 1. Assay quality in each experimental step

The Z'-factor of 0.569 (N = 52 positive controls and N = 70 negative controls) indicated that across all three experimental steps (without experimental sets retesting the results in SARS-CoV-2 variants), the separation between the negative and positive controls was sufficient to perform an 'excellent' assay[1].

The quality of the in vitro assay for characterizing the monotherapies was 'excellent' as indicated by Z'- factor of 0.702 (N = 24). No effects of the vehicle on the %Inhibition and %Cytotoxicity were detected when the maximum used vehicle concentration (0.1% DMSO) was compared with the media only cell controls (Student's two-tailed t-test, N = 24, P = 0.628 and P = 0.092, respectively). We observed that cytotoxicity >25% had effects on the inhibition assay result, therefore we excluded the %Inhibition data points that had >25% corresponding %Cytotoxicity values.

The condensed experimental set had a Z'-factor of 0.590 (N = 12 positive control replicates and N = 18 negative control replicates, respectively), indicating an 'excellent' assay quality. No effect of the vehicle (0.001% DMSO controls) was detected on the %Inhibition and %Cytotoxicity (Wilcoxon rank-sum test, N = 9 vehicle controls and 9 cells in media controls, P = 1 and 1, respectively). Box-Cox transformation was performed on %Inhibition and %Cytotoxicity data, and a cubic transformation (T^3) was applied to improve the residual distributions and the quadratic equations fit, represented by the adjusted R^2 value (Supplementary Figures 6 and 7). In the performed outlier analysis, we accepted a substantial variation of the data and as such no data point was excluded to represent biological and experimental variation.

The validation experimental set with the propagated SARS-CoV-2 variant had a 'do-able' assay quality as indicated by the Z'-factor of 0.408 (N = 16 positive control replicates and 28

negative control replicates, respectively). No effect of the vehicle (0.006% DMSO controls) was detected on the %Inhibition and %Cytotoxicity in Vero E6 cells (Wilcoxon rank-sum test, N = 15 vehicle controls and 13 cells in media controls, P = 1 and 0.711 respectively). None of the %Inhibition data points were excluded based on the %Cytotoxicity as we did not observe the value drop pattern in %Inhibition that would have correlated with %Cytotoxicity indicating the inhibition assay results were not affected by the cytotoxic effects of the drugs. No effect of the vehicle (0.006% DMSO controls) was detected on the %Cytotoxicity in THLE-2 and AC16 cells (Wilcoxon rank-sum test, N = 18, P = 1 and 0.142 respective).

The assay quality of the validation experimental sets with the SARS-CoV-2 B.1.351 and B.1.617.2 variants was ‘excellent’ as indicated by the Z’-factor of 0.576 and 0.503, respectively (N = 8 positive control replicates and 12 negative control replicates for experimental set for each variant). No effect of the vehicle (0.002% DMSO controls) was detected on the %Inhibition (Wilcoxon rank-sum test, N = 8 vehicle controls and N = 10 cells in media control, P = 0.302 and 0.075 respectively). No data points were excluded for the subsequent analyses.

Supplementary Note 2. C_{max} selection

If extensive C_{max} information was available in the literature, the selection was determined by: i) C_{max} resulting from the doses that obtained regulatory approvals, ii) C_{max} at steady state to reflect the drug availability during the multiday SARS-CoV-2 treatment, iii) additionally, if available, we considered the emerging dosages and dosing schedules of the drugs for SARS-CoV-2 treatment as derived from the registered clinical trials. The specifics of the C_{max} selection for each drug is presented below.

Following oral administration of 800 mg bid EIDD-2801 for 5.5 days, its active metabolite EIDD-1931 reached a steady-state C_{max} of 2970 ng/mL (11.457 μM)[2]. BRT given at 4 mg qd achieved a C_{max} of 52 ng/mL (0.140 μM)[3]. EBS given orally at 600 mg twice daily (bid) for 4

days had a reported C_{max} of 0.372 ng/mL (0.00136 μM)[4]. The FDA label reported C_{max} of SEL was 540 ng/mL (1.218 μM) following multiple doses of 80 mg on day 1 and 3 of each week for 2 weeks[5]. The steady-state C_{max} of MST was 264 ng/mL (0.529 μM) when administered orally at 200 mg once daily (qd) for 7 days[6]. NFM had a reported C_{max} of 130 ng/mL (0.241 μM) when administered intravenously at 0.2 mg/kg/h for 13 to 23 days[7]. The reported C_{max} for TPV was 3510 ng/mL (5.163 μM) after multiple oral doses of 750 mg once every 8 hours with peginterferon alfa and ribavirin in accordance with the FDA label[8]. SN-38 had a reported C_{max} of 56 ng/mL (0.143 μM) when a single 340 mg/m^2 irinotecan dose was administered intravenously[9]. A single oral dose of 400 mg IMT resulted in a C_{max} of 1606 ng/mL (2.723 μM)[10]. According to a recent update by the Food and Drug Administration, RDV achieved a steady-state C_{max} of 2229 ng/mL (3.699 μM) when given intravenously at a 200 mg loading dose followed by 100 mg for 4 or 9 days[11]. Notably, this steady-state C_{max} is lower than the C_{max} after a single 200 mg dose (9.0 μM) used in the IDentif.AI study in 2020[12]. This change reflects the learnings in the one year from the pandemic emergence. LPV was used in combination with RTV at 400/100 mg bid, with a steady-state C_{max} of 12300 ng/mL (19.561 μM) after 2 weeks[13]. The reported C_{max} for RTV after a single dose of 600 mg was 14700 ng/mL (20·390 μM)[14].

Supplementary Note 3. %Cytotoxicity in the IDentif.AI-x experimental step

IDentif.AI-x analysis also evaluated %Cytotoxicity of the drug combinations to determine their safety. The resulting adjusted R^2 value was 0.0173 indicating that a quadratic equation did not have an appropriate fit to describe the %Cytotoxicity in this interaction space (Supplementary Table 3 and Supplementary Figure 7). This is attributed to an insufficient cytotoxicity effect detected (< 10%Cytotoxicity) when compared to the variation of measurements done in triplicates (average propagated s.d. = 8.9%) that is inappropriate to perform the analysis with IDentif.AI-x.

2. Supplementary References

1. Zhang, J. H., Chung, T. D. Y. & Oldenburg, K. R. A simple statistical parameter for use in evaluation and validation of high throughput screening assays. *J. Biomol. Screen.* **4**, 67–73 (1999).
2. Painter, W. P. et al. Human Safety, Tolerability, and Pharmacokinetics of Molnupiravir, a Novel Broad-Spectrum Oral Antiviral Agent with Activity Against SARS-CoV-2. Antimicrob Agents Chemother, doi:10.1128/AAC.02428-20 (2021).
3. FDA. Olumiant: Pharmacology/Toxicology Review. https://www.accessdata.fda.gov/drugsatfda_docs/nda/2018/207924Orig1s000PharmR.pdf (2018).
4. Kil, J. et al. Safety and efficacy of ebselen for the prevention of noise-induced hearing loss: a randomised, double-blind, placebo-controlled, phase 2 trial. Lancet 390, 969-979, doi:10.1016/S0140-6736(17)31791-9 (2017).
5. FDA. Selinexor: Multi-Discipline Review https://www.accessdata.fda.gov/drugsatfda_docs/nda/2019/212306Orig1s000MultidisciplineR.pdf (2018).
6. EMA. Masipro: Assessment Report. https://www.ema.europa.eu/en/documents/assessment-report/masipro-epar-refusal-public-assessment-report_en.pdf (2017).
7. KEGG. Fusan (Nafamostat Mesilate). https://www.kegg.jp/medicus-bin/japic_med?japic_code=00048710 (2019).
8. FDA. INCIVEK. https://www.accessdata.fda.gov/drugsatfda_docs/label/2011/201917lbl.pdf (2011).
9. FDA. Camptosar (irinotecan hydrochloride injection). https://www.accessdata.fda.gov/drugsatfda_docs/label/2002/20571s16lbl.pdf (2002).
10. Nikolova, Z. et al. Bioequivalence, safety, and tolerability of imatinib tablets compared with capsules. Cancer Chemother Pharmacol 53, 433-438, doi:10.1007/s00280-003-0756-z (2004).

11. FDA. Veklury (remdesdivir) EUA Fact Sheet for Healthcare Providers. https://www.fda.gov/media/137566/download (2020).
12. Blasiak, A. et al. IDentif.AI: Rapidly optimizing combination therapy design against severe Acute Respiratory Syndrome Coronavirus 2 (SARS-Cov-2) with digital drug development. Bioeng Transl Med 6, e10196, doi:10.1002/btm2.10196 (2021).
13. EMA. Kaletra: Summary of Product Characteristics. https://www.ema.europa.eu/en/documents/product-information/kaletra-epar-product-information_en.pdf (2011).
14. EMA. Norvir: European Public Assessment Preport - Scientific Discussion. https://www.ema.europa.eu/en/documents/scientific-discussion/norvir-epar-scientific-discussion_en.pdf (2005).

3. Supplementary Figures

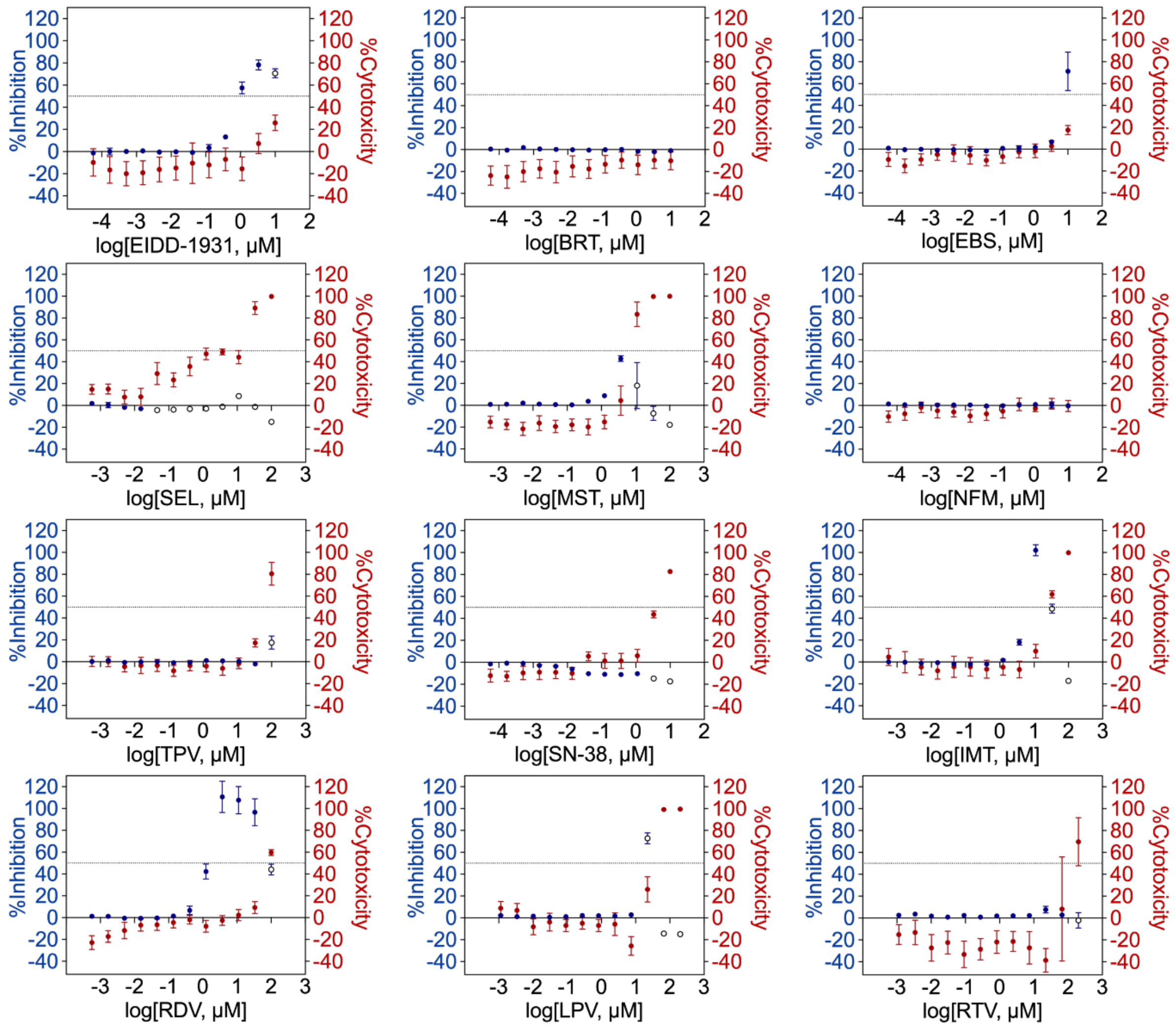

Supplementary Figure 1. Dose-response curves for all 12 selected drugs given as monotherapies. Vero E6 cells infected with SARS-CoV-2 at 100 $TCID_{50}$ were treated with each drug at different concentrations for 72 h. The viral infection %Inhibition and %Cytotoxicity resulted from these drug treatments in the Vero E6 cells were determined by measuring the luminescence signals of the cell viability in the ATP activity assay. The left and right y-axis represent the %Inhibition (blue) and %Cytotoxicity (red) of the drugs, respectively. The unfilled circles (blue) in the dose-response curves were %Inhibition data points with corresponding toxicities above 25% and were excluded from subsequent dose-response curve analysis. Dotted lines at 50% inhibition and 50% cytotoxicity levels represent absolute EC_{50} and CC_{50}, respectively. Data points are mean ± propagated s.d. (N = 3).

Baricitinib (BRT), ebselen (EBS), selinexor (SEL), masitinib (MST), nafamostat mesylate (NFM), telaprevir (VX-950) (TPV), imatinib mesylate (IMT), remdesivir (RDV), lopinavir (LPV), and ritonavir (RTV). The data underlying the graphs can be found in Supplementary Data 3.

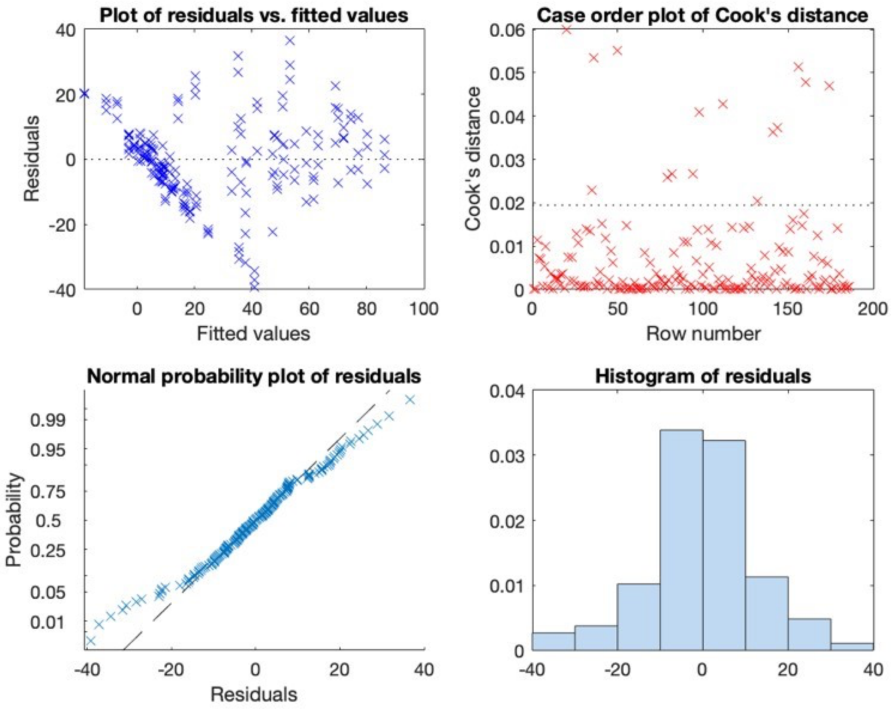

Supplementary Figure 2. Outlier analysis for individual replicates in IDentif.AI-x %Inhibition analysis. All experimental replicates (N = 3) of the %Inhibition of OACD-designed combinations and drug monotherapies were used in a quadratic stepwise regression analysis. Residuals represent the difference between the experimentally determined %Inhibition and the IDentif.AI-x-determined %Inhibition. The plot of residuals vs. fitted values examined the distributions of residuals and the quadratic model fit. Row number in the Cook's distance plot represents each OACD combination and monotherapy (triplicates) in order. The normal probability plot and the histogram of residuals were used to assess the normality of residual distribution. No data points were removed for the IDentif.AI-x analysis.

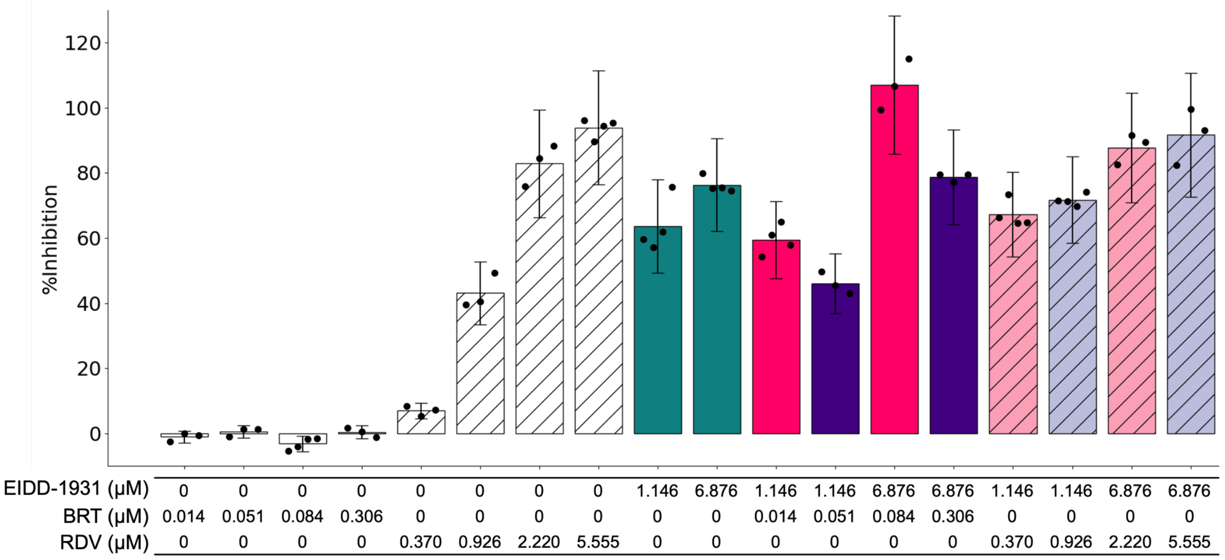

Supplementary Figure 3. Validation of EIDD-1931 interactions at 10% and 60%C_{max} with remdesivir (RDV), and baricitinib (BRT). EIDD-1931 (green), BRT (white), RDV (white, patterned) and EIDD-1931 combinations at the C_{max} ratio (pink) and at the OACD ratio (purple) at different concentrations were added to Vero E6 cells infected with the propagated, original SARS-CoV-2 strain at 100 $TCID_{50}$ and incubated for 72 h. The %Inhibition resulted from the treatments were measured via the luminescence signals of the cell viability in the ATP activity assay. Data points are presented as mean ± propagated s.d. (N = 3 to 4 replicates). Of note, this propagated s.d. did not arise from the replicates, but from plate-to-plate variation from control s.d. Black, round markers indicate individual replicates. No statistically significant difference was detected with Kruskal-Wallis test when followed by Dunn's post hoc test.

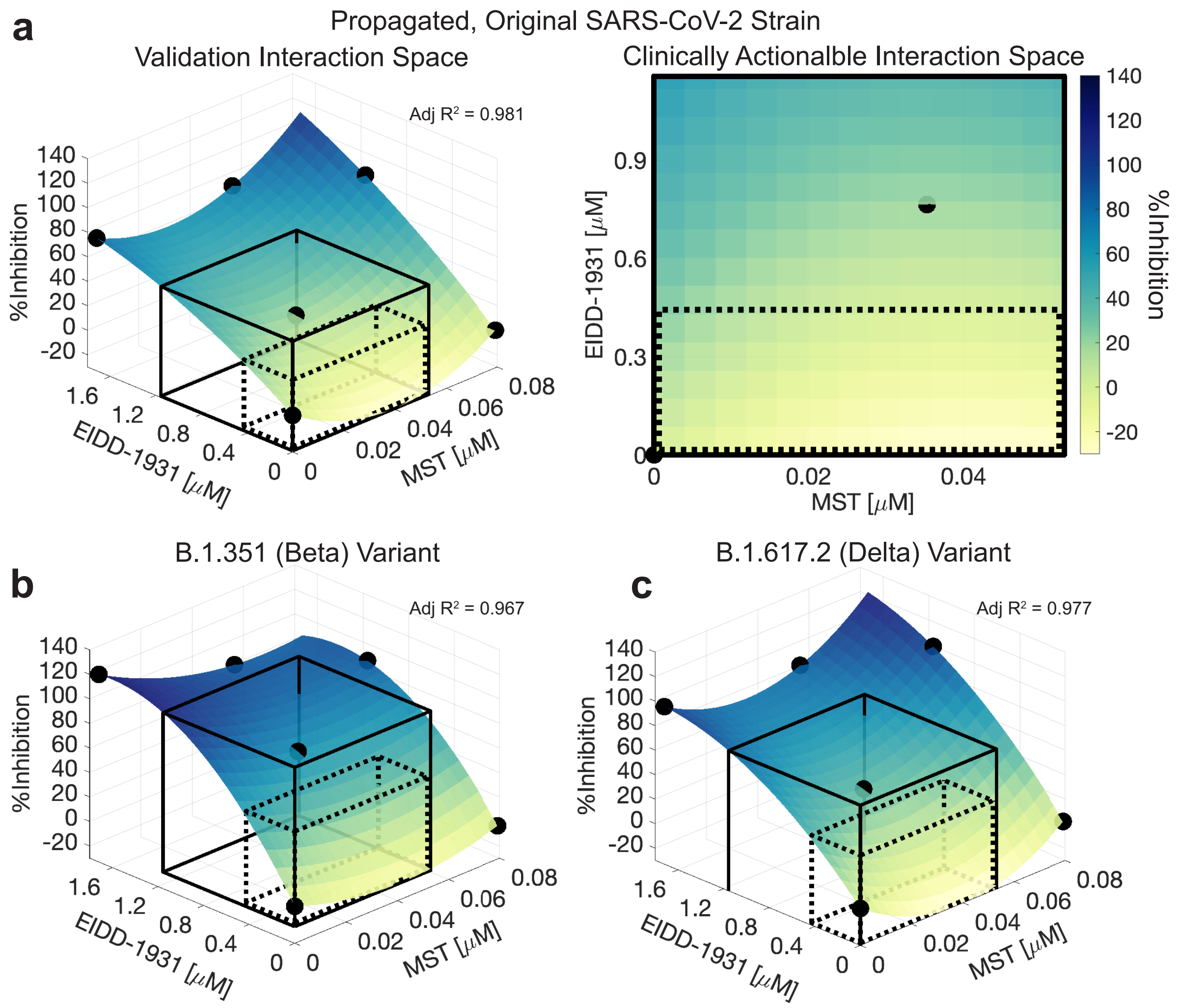

Supplementary Figure 4. Validation of EIDD-1931 interaction with masitinib (MST) (**a**) Surface plot of EIDD-1931/MST %Inhibition activity against propagated, original SARS-CoV-2 strain in the validation interaction space, clinically actionable interaction space (black, solid line border) and the interaction space from the IDentif.AI-x analysis (black, dotted line border). The latter 2 are also shown as a 2-dimensional map. (**b,c**) Surface plot of EIDD-1931/MST interaction against B.1.351 and B.1.617.2 SARS-CoV-2 variants. All experiments were performed with N = 3 to 4 replicates, which were independently included in the surface construction. Black, round markers indicate an average %Inhibition of the replicates for each treatment. Adjusted R^2 (Adj R^2) indicates goodness of the fit for each interaction surface.

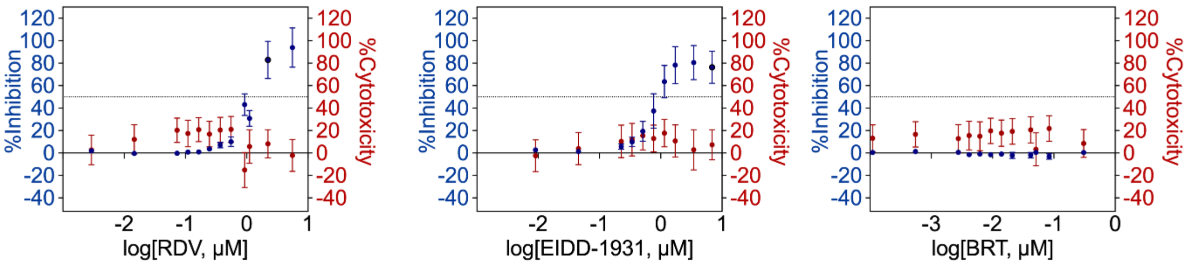

Supplementary Figure 5. Validation dose-response analysis for remdesivir (RDV), EIDD-1931, and baricitinib (BRT). RDV, EIDD-1931, and BRT at different concentrations were added to Vero E6 cells infected with SARS-CoV-2 at 100 $TCID_{50}$ and incubated for 72 h. The %Inhibition and %Cytotoxicity resulted from the treatments were measured via the luminescence signals of the cell viability in the ATP activity assay. The left and right y-axis of each curve represent the %Inhibition (blue) and %Cytotoxicity (red) for the drugs, respectively. Dotted lines at 50% inhibition and 50% cytotoxicity levels represent absolute EC_{50} and CC_{50}, respectively. No data points were excluded in dose-response curve analysis. Data points are presented as mean ± propagated s.d. (N = 3 to 4).

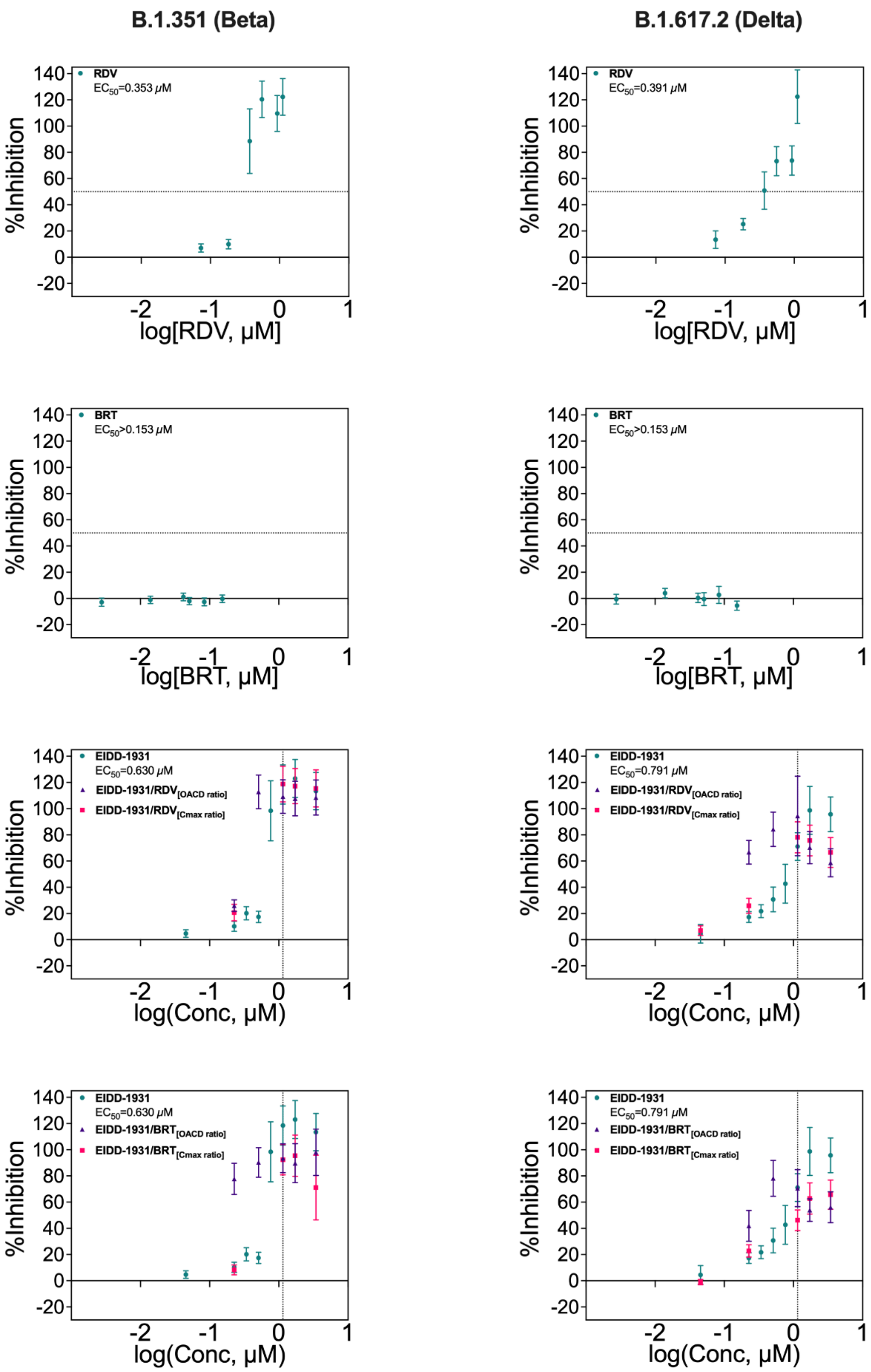

Supplementary Figure 6. Dose-response curves for EIDD-1931, remdesivir (RDV), and baricitinib (BRT) in monotherapies and in combinations against SARS-CoV-2 B.1.351 and B.1.617.2 variants. Increasing concentrations of EIDD-1931, RDV, and BRT

monotherapies were added to Vero E6 cells infected with B.1.351 and B.1.617.2 variants at 100 $TCID_{50}$ and incubated for 72 h. Additionally, selected EIDD-1931/RDV and EIDD-1931/BRT combinations at the OACD and C_{max} ratios (purple and pink markers, respectively) were also added to the infected Vero E6 cells and incubated for 72 h. The %Inhibition resulted from the mono- and combination therapies were measured via the luminescence signals of the cell viability in the ATP activity assay. The y-axis of each curve represent the %Inhibition for the each drug and combination. The absolute EC_{50} values for monotherapies are also summarized in the legends. Vertical dotted lines represent the 10% C_{max} of EIDD-1931, and the horizontal dotted lines at 50% inhibition level represent the absolute EC_{50} for monotherapies. No data points were excluded in dose-response curve analysis. Data points are presented as mean ± propagated s.d. (N = 3 replicates).

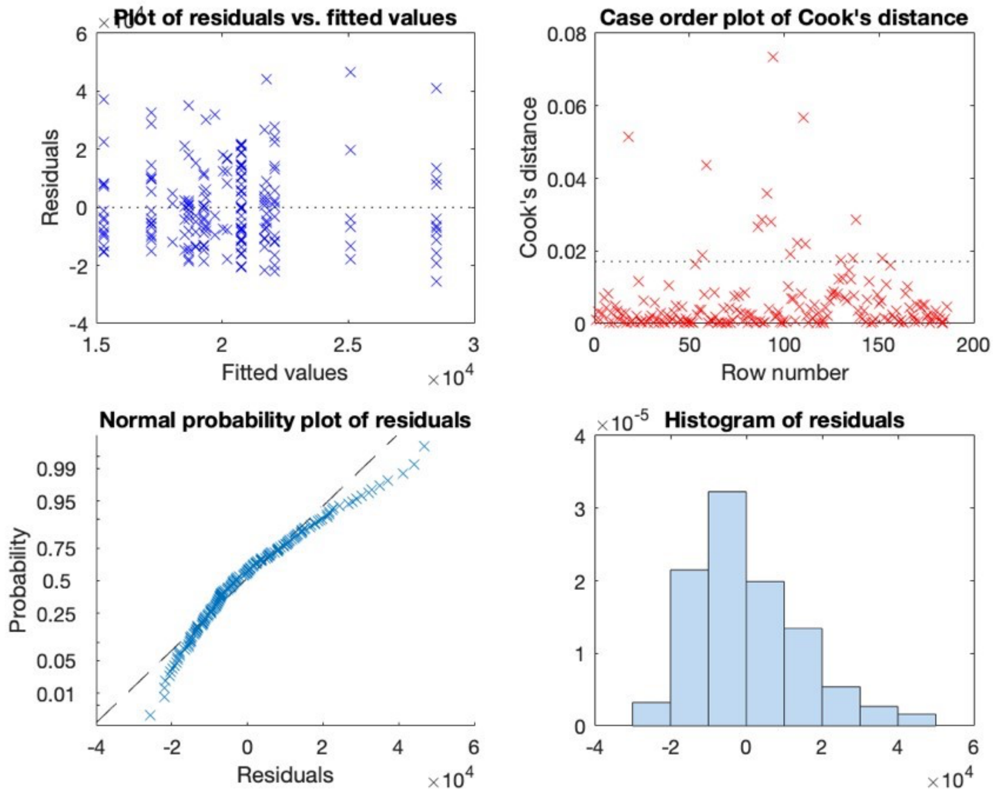

Supplementary Figure 7. Outlier analysis for individual replicates in IDentif.AI-x Vero E6 %Cytotoxicity analysis. All experimental replicates (N = 3) of the %Cytotoxicity of OACD-designed combinations and drug monotherapies were used in a quadratic stepwise regression analysis. Residuals represent the difference between the experimentally determined %Inhibition and the IDentif.AI-x-determined %Inhibition. The plot of residuals vs. fitted values examined the distributions of residuals and the quadratic model fit. Row number in the Cook's distance plot represents each OACD combination and monotherapy (triplicates) in order. The normal probability plot and the histogram of residuals were used to assess the normality of residual distribution. No data points were removed for the IDentif.AI-x analysis.

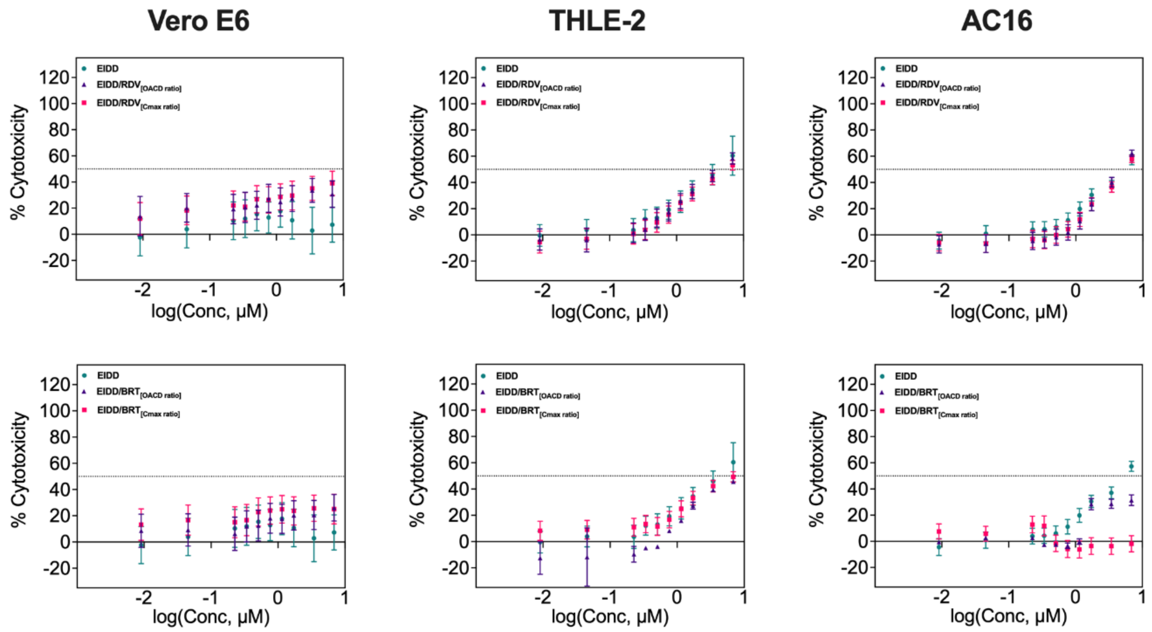

Supplementary Figure 8. %Cytotoxicity data for EIDD-1931/RDV and EIDD-1931/BRT in Vero E6, THLE-2, and AC16 cell lines. %Cytotoxicity for Vero E6 was determined after 72 h of treatment with EIDD-1931/RDV and EIDD-1931/BRT combinations at increasing concentrations using luminescence-based ATP activity assay. %Cytotoxicity data (mean ± propagated s.d., N = 3 to 4 replicates) for EIDD-1931/RDV and EIDD-1931/BRT at two different ratios: OACD ratio (Level 2/Level 2 ratio for EIDD-1931/RDV and Level 1/Level 2 ratio for EIDD-1931/BRT from the IDentif.AI-x experimental set; purple triangles) and C_{max} ratio (C_{max}/C_{max}; pink squares) of the two drugs in the combination. Additionally, %Cytotoxicity was measured in THLE-2 human liver cell lines and AC16 human cardiomyocyte. EIDD-1931/RDV and EIDD-1931/BRT were added to the cells for 72 h before measuring the cell viability via luminescence-based ATP activity assay. Dotted lines at 50% cytotoxicity levels represent absolute CC_{50}. Remdesivir (RDV), and baricitinib (BRT).

4. Supplementary Tables

Supplementary Table 1. Resolution VI six-drug Orthogonal Array Composite Design (OACD) table. 50 combinations for six-drug library at three different concentration levels. Level 0 (input -1 in the OACD table) indicates the absence of a drug. Level 1 and Level 2 (inputs 0 and 1 in the OACD table, respectively) represent two clinically actionable drug concentrations. Remdesivir (RDV), ebselen (EBS), masitinib (MST), imatinib mesylate (IMT), and baricitinib (BRT).

Combination	RDV	EBS	MST	IMT	BRT	EIDD-1931
1	-1	-1	-1	-1	-1	-1
2	1	-1	-1	-1	-1	1
3	-1	1	-1	-1	-1	1
4	1	1	-1	-1	-1	-1
5	-1	-1	1	-1	-1	1
6	1	-1	1	-1	-1	-1
7	-1	1	1	-1	-1	-1
8	1	1	1	-1	-1	1
9	-1	-1	-1	1	-1	1
10	1	-1	-1	1	-1	-1
11	-1	1	-1	1	-1	-1
12	1	1	-1	1	-1	1
13	-1	-1	1	1	-1	-1
14	1	-1	1	1	-1	1
15	-1	1	1	1	-1	1
16	1	1	1	1	-1	-1
17	-1	-1	-1	-1	1	1
18	1	-1	-1	-1	1	-1
19	-1	1	-1	-1	1	-1
20	1	1	-1	-1	1	1

21	-1	-1	1	-1	1	-1
22	1	-1	1	-1	1	1
23	-1	1	1	-1	1	1
24	1	1	1	-1	1	-1
25	-1	-1	-1	1	1	-1
26	1	-1	-1	1	1	1
27	-1	1	-1	1	1	1
28	1	1	-1	1	1	-1
29	-1	-1	1	1	1	1
30	1	-1	1	1	1	-1
31	-1	1	1	1	1	-1
32	1	1	1	1	1	1
33	-1	-1	-1	-1	-1	-1
34	-1	0	0	0	0	0
35	-1	1	1	1	1	1
36	0	-1	-1	0	0	1
37	0	0	0	1	1	-1
38	0	1	1	-1	-1	0
39	1	-1	0	-1	1	0
40	1	0	1	0	-1	1
41	1	1	-1	1	0	-1
42	-1	-1	1	1	0	0
43	-1	0	-1	-1	1	1
44	-1	1	0	0	-1	-1
45	0	-1	0	1	-1	1
46	0	0	1	-1	0	-1
47	0	1	-1	0	1	0
48	1	-1	1	0	1	-1

49	1	0	-1	1	-1	0
50	1	1	0	-1	0	1

Supplementary Table 2. IDentif.AI-x estimated coefficients and modelling statistics for %Inhibition analysis. Remdesivir (RDV), ebselen (EBS), masitinib (MST), imatinib mesylate (IMT), and baricitinib (BRT). Statistical significance was determined using F-test. *P < 0.05, **P < 0.01 and, ***P < 0.001.

	Estimated Coefficients	Statistical Significance
Intercept	21.703	***
RDV	7.458	***
EBS	-4.917	***
MST	-0.043	
IMT	0.011	
BRT	-4.930	***
EIDD-1931	22.654	***
RDV:MST	-3.417	**
RDV:IMT	-1.955	
RDV:EIDD-1931	6.646	***
EBS:MST	-2.465	*
EBS:BRT	-3.878	***
EBS:EIDD-1931	-4.004	***
BRT:EIDD-1931	-4.954	***
MST^2	6.035	
BRT^2	19.688	***
$EIDD\text{-}1931^2$	-16.002	***
**Model Statistics**		
Adjusted R^2 (IDentif.AI-x)		0.794
Number of observations		186
Error degrees of freedom		169

Supplementary Table 3. IDentif.AI-x estimated coefficients and modelling statistics for Vero E6 %Cytotoxicity analysis. Remdesivir (RDV), masitinib (MST), imatinib mesylate (IMT), and baricitinib (BRT). Statistical significance was determined using F-test. *P < 0.05, **P < 0.01, and *** P < 0.001.

	Estimated Coefficients	Statistical Significance
Intercept	20426.0	***
RDV	1460.3	
MST	1076.5	
IMT	1321.9	
RDV:MST	2125.8	
RDV:IMT	2072.3	
**Model statistics**		
Adjusted R^2 (IDentif.AI-x)		0.017
Number of observations		186
Error degrees of freedom		180

5. Supplementary Data 1

MATLAB code, %Inhibition and %Cytotoxicity experimental data for IDentif.AI-x analysis. The code does not include data transformation as it was deemed not required for these datasets. Remdesivir (RDV), ebselen (EBS), masitinib (MST), imatinib mesylate (IMT), and baricitinib (BRT).

```
%%%%%%% 1. LOAD DATA %%%%%%%
% Dataset 1: Load Inhibition response experimental data
data_inhibition = [
    %  RDV       EBS       MST       IMT       BRT  EIDD-1931   %inhibition(3 replicates)
   -1.0000   -1.0000   -1.0000   -1.0000   -1.0000   -1.0000   -1.2397    0.2025    2.9865
    1.0000   -1.0000   -1.0000   -1.0000   -1.0000    1.0000   87.4615   83.3856   92.2759
   -1.0000    1.0000   -1.0000   -1.0000   -1.0000    1.0000   57.5374   44.4547   59.5971
    1.0000    1.0000   -1.0000   -1.0000   -1.0000   -1.0000    6.8992    5.8718    9.3749
   -1.0000   -1.0000    1.0000   -1.0000   -1.0000    1.0000   45.7130   57.7155   67.4991
    1.0000   -1.0000    1.0000   -1.0000   -1.0000   -1.0000    3.3394    0.7584    5.4285
   -1.0000    1.0000    1.0000   -1.0000   -1.0000   -1.0000   -2.1343   -3.0879    2.3004
    1.0000    1.0000    1.0000   -1.0000   -1.0000    1.0000   84.7934   74.1109   91.7061
   -1.0000   -1.0000   -1.0000    1.0000   -1.0000    1.0000   55.6883   46.4223   50.9709
    1.0000   -1.0000   -1.0000    1.0000   -1.0000   -1.0000    8.6950    8.2249   13.2216
   -1.0000    1.0000   -1.0000    1.0000   -1.0000   -1.0000    2.1252    2.0587    2.9345
    1.0000    1.0000   -1.0000    1.0000   -1.0000    1.0000   78.9961   73.8726   89.6854
   -1.0000   -1.0000    1.0000    1.0000   -1.0000   -1.0000    3.5457    4.0890    5.1021
    1.0000   -1.0000    1.0000    1.0000   -1.0000    1.0000   88.0256   72.7613   83.9413
   -1.0000    1.0000    1.0000    1.0000   -1.0000    1.0000   54.9985   43.8653   55.2760
    1.0000    1.0000    1.0000    1.0000   -1.0000   -1.0000    8.9696    9.8203    9.9327
   -1.0000   -1.0000   -1.0000   -1.0000    1.0000    1.0000   44.9842   61.8942   66.8201
    1.0000   -1.0000   -1.0000   -1.0000    1.0000   -1.0000    7.5836    9.9357    6.8326
   -1.0000    1.0000   -1.0000   -1.0000    1.0000   -1.0000    3.3075    2.3185   -0.3022
    1.0000    1.0000   -1.0000   -1.0000    1.0000    1.0000   89.9939   77.8160   82.2708
   -1.0000   -1.0000    1.0000   -1.0000    1.0000   -1.0000    2.9009    1.9052    0.8485
    1.0000   -1.0000    1.0000   -1.0000    1.0000    1.0000   78.3541   78.8751   81.9017
   -1.0000    1.0000    1.0000   -1.0000    1.0000    1.0000    2.7811    5.9182    1.6107
    1.0000    1.0000    1.0000   -1.0000    1.0000   -1.0000    7.1977    9.0210    4.6476
   -1.0000   -1.0000   -1.0000    1.0000    1.0000   -1.0000    3.8357    1.8886    2.3015
    1.0000   -1.0000   -1.0000    1.0000    1.0000    1.0000   85.7306   63.0263   82.9097
   -1.0000    1.0000   -1.0000    1.0000    1.0000    1.0000    2.4606    2.3086    0.2792
    1.0000    1.0000   -1.0000    1.0000    1.0000   -1.0000   12.9480    7.3118    5.1683
   -1.0000   -1.0000    1.0000    1.0000    1.0000    1.0000   55.2297   66.8572   70.1605
    1.0000   -1.0000    1.0000    1.0000    1.0000   -1.0000    7.5081   12.1730    9.8911
   -1.0000    1.0000    1.0000    1.0000    1.0000   -1.0000    1.9896    1.1110    3.8357
    1.0000    1.0000    1.0000    1.0000    1.0000    1.0000   21.1504   14.8315    5.9686
   -1.0000   -1.0000   -1.0000   -1.0000   -1.0000   -1.0000    4.5754    2.9768    0.8151
   -1.0000         0         0         0         0         0   31.9528   26.7425   32.8659
   -1.0000    1.0000    1.0000    1.0000    1.0000    1.0000   45.8499   40.0406   42.5401
         0   -1.0000   -1.0000         0         0    1.0000    1.5727    6.4862    3.6951
         0         0         0    1.0000    1.0000   -1.0000    7.5135    7.7587    3.5426
         0    1.0000    1.0000   -1.0000   -1.0000         0   55.6242   39.4815   40.6570
    1.0000   -1.0000         0   -1.0000    1.0000         0   59.4541   51.7028   48.1089
    1.0000         0    1.0000         0   -1.0000    1.0000   88.5446   71.2516   86.8213
```

```
   1.0000    1.0000   -1.0000    1.0000         0   -1.0000   10.7241    9.9275    5.5841
  -1.0000   -1.0000    1.0000    1.0000         0         0   29.0793   23.3247   35.8803
  -1.0000         0   -1.0000   -1.0000    1.0000    1.0000    2.3939    3.2527    1.7894
  -1.0000    1.0000         0         0   -1.0000   -1.0000    5.0606    4.2987    4.0959
        0   -1.0000         0    1.0000   -1.0000    1.0000   70.4509   61.2460   67.0435
        0         0    1.0000   -1.0000         0   -1.0000    6.4319    7.5852    3.9609
        0    1.0000   -1.0000         0    1.0000         0   48.8782   44.0221   29.2327
   1.0000   -1.0000    1.0000         0    1.0000   -1.0000   10.1778    7.5359    4.9138
   1.0000         0   -1.0000    1.0000   -1.0000         0   56.1318   54.8656   48.9830
   1.0000    1.0000         0   -1.0000         0    1.0000    4.7656    8.4474    7.1520
        0   -1.0000   -1.0000   -1.0000   -1.0000   -1.0000    3.2162    5.6682    4.4560
  -1.0000         0   -1.0000   -1.0000   -1.0000   -1.0000    4.1780    3.0119    2.8291
  -1.0000   -1.0000         0   -1.0000   -1.0000   -1.0000    4.2077    4.8337    4.6648
  -1.0000   -1.0000   -1.0000         0   -1.0000   -1.0000    3.3411    2.7375    3.6500
  -1.0000   -1.0000   -1.0000   -1.0000         0   -1.0000    1.8137    1.5446    1.4202
  -1.0000   -1.0000   -1.0000   -1.0000   -1.0000         0   37.4825   36.9267   27.7308
   1.0000   -1.0000   -1.0000   -1.0000   -1.0000   -1.0000    5.0925    7.2433    7.0131
  -1.0000    1.0000   -1.0000   -1.0000   -1.0000   -1.0000    7.1741    4.9019    1.2521
  -1.0000   -1.0000    1.0000   -1.0000   -1.0000   -1.0000    6.6284    4.3518    4.0419
  -1.0000   -1.0000   -1.0000    1.0000   -1.0000   -1.0000    8.8283    3.8325    0.6762
  -1.0000   -1.0000   -1.0000   -1.0000    1.0000   -1.0000    8.3530    4.3796    0.7792
  -1.0000   -1.0000   -1.0000   -1.0000   -1.0000    1.0000   48.7931   24.6995   41.9429];

% Dataset 2: Load Cytotoxicity response experimental data
data_cytotoxicity = [
    %  RDV       EBS       MST       IMT      BRT  EIDD-1931   %cytotoxicity(3 reps)
  -1.0000   -1.0000   -1.0000   -1.0000   -1.0000   -1.0000    1.0103   12.1434  -18.1770
   1.0000   -1.0000   -1.0000   -1.0000   -1.0000    1.0000   -4.4554    5.4813  -22.7111
  -1.0000    1.0000   -1.0000   -1.0000   -1.0000    1.0000    1.1566    4.5936  -15.6860
   1.0000    1.0000   -1.0000   -1.0000   -1.0000   -1.0000   -3.8146   -0.0928  -15.8575
  -1.0000   -1.0000    1.0000   -1.0000   -1.0000    1.0000   -6.1358    4.1898  -17.4153
   1.0000   -1.0000    1.0000   -1.0000   -1.0000   -1.0000   -0.6103   -0.3434  -19.7204
  -1.0000    1.0000    1.0000   -1.0000   -1.0000   -1.0000   -7.4185    2.7223  -21.2843
   1.0000    1.0000    1.0000   -1.0000   -1.0000    1.0000    6.2589    5.3861   -2.8199
  -1.0000   -1.0000   -1.0000    1.0000   -1.0000    1.0000   -0.9267    8.4767   -4.4217
   1.0000   -1.0000   -1.0000    1.0000   -1.0000   -1.0000    6.2192    5.9973   -9.6750
  -1.0000    1.0000   -1.0000    1.0000   -1.0000   -1.0000    8.6779    6.3587  -13.3385
   1.0000    1.0000   -1.0000    1.0000   -1.0000    1.0000    0.3845   10.3406  -18.0095
  -1.0000   -1.0000    1.0000    1.0000   -1.0000   -1.0000    5.5657    7.5587   -9.9034
   1.0000   -1.0000    1.0000    1.0000   -1.0000    1.0000   10.4254   12.0006   -8.4960
  -1.0000    1.0000    1.0000    1.0000   -1.0000    1.0000    0.9799    7.1619   -3.6405
   1.0000    1.0000    1.0000    1.0000   -1.0000   -1.0000    4.4807    6.3159    5.3932
  -1.0000   -1.0000   -1.0000   -1.0000    1.0000    1.0000    3.4912   12.1625    0.4196
   1.0000   -1.0000   -1.0000   -1.0000    1.0000   -1.0000   14.7018    5.9508   -5.0536
  -1.0000    1.0000   -1.0000   -1.0000    1.0000   -1.0000    6.9282    4.7976    2.2082
   1.0000    1.0000   -1.0000   -1.0000    1.0000    1.0000   -0.3957    5.8568  -10.2699
  -1.0000   -1.0000    1.0000   -1.0000    1.0000   -1.0000    4.1304   -0.6767  -14.0428
   1.0000   -1.0000    1.0000   -1.0000    1.0000    1.0000    1.5957    6.6487    5.8381
  -1.0000    1.0000    1.0000   -1.0000    1.0000    1.0000   10.4897    3.3901    1.8623
   1.0000    1.0000    1.0000   -1.0000    1.0000   -1.0000    6.6415   13.7384    7.5668
  -1.0000   -1.0000   -1.0000    1.0000    1.0000   -1.0000    2.3485    1.9202   -6.3631
   1.0000   -1.0000   -1.0000    1.0000    1.0000    1.0000   10.0121   14.0922   -1.1398
  -1.0000    1.0000   -1.0000    1.0000    1.0000    1.0000    1.8880    4.3975   -4.6782
```

```
    1.0000    1.0000   -1.0000    1.0000    1.0000   -1.0000    5.4218    3.9646  -18.5044
   -1.0000   -1.0000    1.0000    1.0000    1.0000    1.0000   -0.2540   14.1030   -0.9794
    1.0000   -1.0000    1.0000    1.0000    1.0000   -1.0000    5.0839   10.9240    0.6957
   -1.0000    1.0000    1.0000    1.0000    1.0000   -1.0000    2.4552   13.1482    0.1377
    1.0000    1.0000    1.0000    1.0000    1.0000    1.0000    3.0529   18.3969   -1.7235
   -1.0000   -1.0000   -1.0000   -1.0000   -1.0000   -1.0000    7.8917    8.7074   -1.7227
   -1.0000         0         0         0         0         0    1.3355    9.6979   -4.7670
   -1.0000    1.0000    1.0000    1.0000    1.0000    1.0000   -3.0380    7.3443   -3.7549
         0   -1.0000   -1.0000         0         0    1.0000    0.8034    0.2502    6.7577
         0         0         0    1.0000    1.0000   -1.0000    3.0135    4.1747    1.8256
         0    1.0000    1.0000   -1.0000   -1.0000         0    9.1979    0.3534    7.7679
    1.0000   -1.0000         0   -1.0000    1.0000         0   11.3918    1.4536    3.7577
    1.0000         0    1.0000         0   -1.0000    1.0000    4.9743   12.8349   -3.3487
    1.0000    1.0000   -1.0000    1.0000         0   -1.0000    7.6375   12.7727   -5.0967
   -1.0000   -1.0000    1.0000    1.0000         0         0   -3.0249    8.8776   -4.8144
   -1.0000         0   -1.0000   -1.0000    1.0000    1.0000   -6.1111   11.4659   -1.5197
   -1.0000    1.0000         0         0   -1.0000   -1.0000    2.4361   -0.7446   -5.1982
         0   -1.0000         0    1.0000   -1.0000    1.0000    8.5363   17.6671    2.7196
         0         0    1.0000   -1.0000         0   -1.0000   10.5322   10.6272   -9.5861
         0    1.0000   -1.0000         0    1.0000         0    5.6149   14.0076   -0.2522
    1.0000   -1.0000    1.0000         0    1.0000   -1.0000    3.6106   18.8380    0.0687
    1.0000         0   -1.0000    1.0000   -1.0000         0    3.9649   13.2111   -0.8914
    1.0000    1.0000         0   -1.0000         0    1.0000    4.7625   -1.9359   -7.1950
         0   -1.0000   -1.0000   -1.0000   -1.0000   -1.0000    5.5953    4.1756   -4.3038
   -1.0000         0   -1.0000   -1.0000   -1.0000   -1.0000    4.7274   10.2411   -1.8541
   -1.0000   -1.0000         0   -1.0000   -1.0000   -1.0000   14.5474   -1.0824    2.8458
   -1.0000   -1.0000   -1.0000         0   -1.0000   -1.0000    9.2153   10.8582    0.6293
   -1.0000   -1.0000   -1.0000   -1.0000         0   -1.0000   10.0287    6.2955   -1.3376
   -1.0000   -1.0000   -1.0000   -1.0000   -1.0000         0   10.0242    9.5346   -3.4946
    1.0000   -1.0000   -1.0000   -1.0000   -1.0000   -1.0000   10.8039    1.4750   -2.2665
   -1.0000    1.0000   -1.0000   -1.0000   -1.0000   -1.0000   11.9686    0.8058    0.6939
   -1.0000   -1.0000    1.0000   -1.0000   -1.0000   -1.0000   14.9943    4.8994    4.5126
   -1.0000   -1.0000   -1.0000    1.0000   -1.0000   -1.0000    3.7909    8.4376    4.1952
   -1.0000   -1.0000   -1.0000   -1.0000    1.0000   -1.0000    0.9041    8.8292    0.7686
   -1.0000   -1.0000   -1.0000   -1.0000   -1.0000    1.0000    7.1567   11.3136   -5.4645]

%%%%%%% 2. DEFINE INPUTS AND OUTPUTS %%%%%%%
x = data_inhibition(:,1:6); %substitute %cytotoxicity data for %cytotoxicity surface
y = data_inhibition(:,7:9); %substitute %cytotoxicity data for %cytotoxicity surface
x = [x;x;x]; %for 3 replicates
y = y(:);

%%%%%%% 3. GENERATE IDentif.AI QUADRATIC SERIES %%%%%%%
mdl = stepwiselm(x,y,'quadratic', ‘Criterion’, ‘sse’,
'ResponseVar','%Inhibition','PredictorVars',{'RDV', 'EBS', 'MST', 'IMT', 'BRT', 'EIDD-
1931'}); %substitute %Cytotoxicity label %cytotoxicity surface
```

Supplementary Data 2

The complete results of the IDentif.AI analysis. Concentration level of each drug refers to the concentration levels 0, 1, 2 as listed in Table 2.

[See seperete file ‘Supplementary Data 2’]

Supplementary Data 3

The data underlying the monotherapy experimental results shown in Supplementary Figure 1. Three experiemntal replicates for %Inhibition and %Cytotoxicity data are shown as ‘inhib 1-3’ and ‘tox 1-3’ respectively. ‘inhib/tox_avg’ and ‘inhib/tox_sd’ represent an average and a propagated s.d., respectively. Baricitinib (BRT), ebselen (EBS), selinexor (SEL), masitinib (MST), nafamostat mesylate (NFM), telaprevir (VX-950) (TPV), imatinib mesylate (IMT), remdesivir (RDV), lopinavir (LPV), and ritonavir (RTV).

[See seperete file ‘Supplementary Data 3’]

Supplementary Data 4

The data underlying the validation experimental results in Figures 3 to 5. %Inhibition for original, propagated, B.1.351 (Beta) and B.1.617.2 (Delta) SARS-CoV-2 virus strains was derived from the experimental testing in Vero E6. %Cytotoxicity was derived from the experimental testing in Vero E6, THLE-2 and AC16 cell lines. Baricitinib (BRT), ebselen (EBS), masitinib (MST), remdesivir (RDV), lopinavir (LPV), and ritonavir (RTV).

[See seperete file ‘Supplementary Data 4’]
