# Supplementary material for: The IDentif.AI-x pandemic readiness platform: Rapid prioritization of optimized COVID-19 combination therapy regimens
Source: NPJ Digit Med. 2022 Jun 30;5:83. doi: 10.1038/s41746-022-00627-4 (PMC9244889; doi:10.1038/s41746-022-00627-4)
Supplement: Supplementary file 5 — Reporting Summary [file 41746_2022_627_MOESM5_ESM.pdf]

nature portfolio

Corresponding author(s): Agata Blasiak, Conrad En Zuo Chan, Edward Kai-Hua Chow, Dean Ho

Last updated by author(s): 23/05/2022

Reporting Summary

Nature Portfolio wishes to improve the reproducibility of the work that we publish. This form provides structure for consistency and transparency in reporting. For further information on Nature Portfolio policies, see our Editorial Policies and the Editorial Policy Checklist.

Statistics

For all statistical analyses, confirm that the following items are present in the figure legend, table legend, main text, or Methods section.

n/a	Confirmed	
☐	☒	The exact sample size (*n*) for each experimental group/condition, given as a discrete number and unit of measurement
☐	☒	A statement on whether measurements were taken from distinct samples or whether the same sample was measured repeatedly
☐	☒	The statistical test(s) used AND whether they are one- or two-sided<br>*Only common tests should be described solely by name; describe more complex techniques in the Methods section.*
☒	☐	A description of all covariates tested
☐	☒	A description of any assumptions or corrections, such as tests of normality and adjustment for multiple comparisons
☐	☒	A full description of the statistical parameters including central tendency (e.g. means) or other basic estimates (e.g. regression coefficient) AND variation (e.g. standard deviation) or associated estimates of uncertainty (e.g. confidence intervals)
☐	☒	For null hypothesis testing, the test statistic (e.g. *F*, *t*, *r*) with confidence intervals, effect sizes, degrees of freedom and *P* value noted<br>*Give P values as exact values whenever suitable.*
☒	☐	For Bayesian analysis, information on the choice of priors and Markov chain Monte Carlo settings
☒	☐	For hierarchical and complex designs, identification of the appropriate level for tests and full reporting of outcomes
☒	☐	Estimates of effect sizes (e.g. Cohen's *d*, Pearson's *r*), indicating how they were calculated

Our web collection on statistics for biologists contains articles on many of the points above.

Software and code

Policy information about availability of computer code

Data collection	JANUS G3 Mini built in software (WinPREP); Tecan micorplate readers built in softwares
Data analysis	Data processing and IDentif.AI-x analyses in this study were conducted using basic functions written in Python programming language and in Matlab R2020a (Mathworks, Inc.). IDentif.AI-x analyses were conducted using the built-in “stepwiselm” function in Matlab R2020a as provided in Supplementary Data 1. The custom codes can be shared on reasonable request to the corresponding authors.

For manuscripts utilizing custom algorithms or software that are central to the research but not yet described in published literature, software must be made available to editors and reviewers. We strongly encourage code deposition in a community repository (e.g. GitHub). See the Nature Portfolio guidelines for submitting code & software for further information.

Data

Policy information about availability of data

All manuscripts must include a data availability statement. This statement should provide the following information, where applicable:

- Accession codes, unique identifiers, or web links for publicly available datasets
- A description of any restrictions on data availability
- For clinical datasets or third party data, please ensure that the statement adheres to our policy

All data generated and analyzed during this study are included in this published article and its supplementary information. The %Inhibition and %Cytotoxicity data together with the code for the 6-drug IDentif.AI-x drug combination optimization step, can be found in the Supplementary Data 1. All %Inhibition and %Cytotoxicity predictions generated in IDentif.AI analysis can be found in Supplementary Data 2. The experimental data underlying the monotherapy and validation analyses can be found in Supplementary Data 3 and 4, respectively.

Field-specific reporting

Please select the one below that is the best fit for your research. If you are not sure, read the appropriate sections before making your selection.

☒ Life sciences ☐ Behavioural & social sciences ☐ Ecological, evolutionary & environmental sciences

For a reference copy of the document with all sections, see nature.com/documents/nr-reporting-summary-flat.pdf

Life sciences study design

All studies must disclose on these points even when the disclosure is negative.

Sample size	All in vitro cellular experiments were performed in at least 3 biological replicates as it is established in the field.<br><br>IMPORTANT NOTE: This information is already included in the submitted manuscript.
Data exclusions	In the Monotherapy dose-response curve analysis, we observed that %cytotoxicity >25% had effects on the inhibition assay result, therefore we excluded the %Inhibition data points that had >25% corresponding %Cytotoxicity values (Excluded data are represented by unfilled blue circles in Supplementary Fig S1). No other data were excluded in other analysis.<br><br>IMPORTANT NOTE: This information is already included in the submitted manuscript.
Replication	All cellular experimental treatments were performed in at least 3 biological replicates. We do not expect any issues with the reproducibility of the results beyond a limited access to the used viral sample.
Randomization	No randomization was performed.
Blinding	The laboratory staff performing the experimental work on viral inhibition was blinded to the well content arrangement on the plates.<br><br>IMPORTANT NOTE: This information is already included in the submitted manuscript.

Reporting for specific materials, systems and methods

We require information from authors about some types of materials, experimental systems and methods used in many studies. Here, indicate whether each material, system or method listed is relevant to your study. If you are not sure if a list item applies to your research, read the appropriate section before selecting a response.

Materials & experimental systems

n/a	Involved in the study	
☒	☐	Antibodies
☐	☒	Eukaryotic cell lines
☒	☐	Palaeontology and archaeology
☐	☒	Animals and other organisms
☒	☐	Human research participants
☒	☐	Clinical data
☒	☐	Dual use research of concern

Methods

n/a	Involved in the study	
☒	☐	ChIP-seq
☒	☐	Flow cytometry
☒	☐	MRI-based neuroimaging

Eukaryotic cell lines

Policy information about cell lines

Cell line source(s)	THLE-2 cells: CRL2706, ATCC; AC16 cells: SCC-09, Millipore; Vero E6 cells C1008
Authentication	None of the cell lines were authenticated
Mycoplasma contamination	The cell lines were not tested for mycoplasma contamination
Commonly misidentified lines<br>(See ICLAC register)	No commonly misidentified cell lines were used

Animals and other organisms

Policy information about studies involving animals; ARRIVE guidelines recommended for reporting animal research

Laboratory animals	No laboratory animals were used
Wild animals	No wild animals were used
Field-collected samples	Severe acute respiratory syndrome coronavirus 2 (SARS-CoV-2) was previously isolated from a nasopharyngeal swab in early 2020 in Singapore as described before and has undergone several rounds of propagation to form SARS-CoV-2 original, propagated variant used in this study (virus source: Biological Defence Programme, DSO National Laboratories). This propagated variant was identified to have genetic mutations as compared to the original strain. The second viral strain – the B.1.351 (Beta) variant – was isolated from a nasopharyngeal swab in early 2021 in Singapore and was registered in GISAID EpiFlu™ Database under hCoV-19/Singapore/239/2021 (virus source: National Public Health Laboratory, NCID). The third viral strain – the B.1.617.2. (Delta) variant – was isolated from a nasopharyngeal swab in 2021 in Singapore (virus source: National Public Health Laboratory, NCID).<br><br>IMPORTANT NOTE: This information is already included in the submitted manuscript
Ethics oversight	NA

Note that full information on the approval of the study protocol must also be provided in the manuscript.
